# A Design of Overlapped Chunked Code over Compute-and-Forward for Multi-Source Multi-Relay Networks†

**DOI:** 10.3390/s18103225

**Published:** 2018-09-25

**Authors:** Rithea Ngeth, Brian M. Kurkoski, Yuto Lim, Yasuo Tan

**Affiliations:** School of Information Science, Japan Advanced Institute of Science and Technology, Nomi, Ishikawa 923-1292, Japan; kurkoski@jaist.ac.jp (B.M.K.), ylim@jaist.ac.jp (Y.L.), ytan@jaist.ac.jp (Y.T.)

**Keywords:** overlapped chunked code, compute-and-forward, nested lattice code, multi-source multi-relay, empirical rank distribution, decodability

## Abstract

This paper investigates the design of overlapped chunked codes (OCC) for multi-source multi-relay networks where a physical-layer network coding approach, compute-and-forward (CF) based on nested lattice codes (NLC), is applied for the simultaneous transmissions from the sources to the relays. This code is called OCC/CF. In this paper, OCC is applied before NLC before transmitting for each source. Random linear network coding is applied within each chunk. A decodability condition to design OCC/CF is provided. In addition, an OCC with a contiguously overlapping, but non-rounded-end fashion is employed for the design, which is done by using the probability distributions of the number of innovative codeword combinations and the probability distribution of the participation factor of each source to the codeword combinations received for a chunk transmission. An estimation is done to select an allocation, i.e., the number of innovative blocks per chunk and the number of blocks taken from the previous chunk for all sources, that is expected to provide the desired performance. From the numerical results, the design overhead of OCC/CF is low when the probability distribution of the participation factor of each source is dense at the chunk size for each source.

## 1. Introduction

This paper is an extended version of the work in [1].

In the current situation, wireless network nodes are ubiquitous and have increasing density. Since the wireless channel bandwidth is limited, the interference between nodes can affect the data transmission between nodes, e.g., causing message loss, longer latency, high energy consumption, etc. In order to solve this issue, the handshaking mechanism is applied to share the channel between nodes via a control message, e.g., request-to-send and clear-to-send. In addition, the transmit power control approach can allow multiple sources to transmit their messages simultaneously with appropriate interference between nodes. On the other hand, some proposed solutions exploited the interference instead of dealing with (or compensating) it. One of these solutions is physical-layer network coding (PNC) [2], which can also allow multiple sources to transmit their messages to the common receivers simultaneously.

In the packet switching network, a message file is divided into small packets or blocks. Network coding is a scheme where the outgoing block of the sender is a function of incoming blocks. By considering the form of data, network coding can be divided into straightforward network coding (SNC) and PNC. Straightforward network coding works with binary or with a symbol in the finite field, and PNC works with signal forms, i.e., with real values. Exclusive or (XOR) is a simple method of SNC. It was applied in a two-way relay channel with inter-flow network coding approach to improve the network throughput and also the energy efficiency [3]. Linear network coding is a general approach of XOR. It linearly combines the incoming blocks in a finite field of size *q*, $F_{q}$ (q=2 for XOR). The combined coefficients are randomly drawn from $F_{q}$ for random linear network coding (RLNC). The application of RLNC was widely studied in wireless multi-hop networks for reliable communication [4,5].

PNC, sometimes called analog network coding, performs additive mixing of received electromagnetic waves (received signals) from different sources naturally at a receiver, then transforms and maps the superimposed received signals into a desired function of transmitted source signals. The time to complete the message transmission can be shorter with PNC if comparing with SNC for example in a two-way relay channel [2], cross atom topology [6], because PNC allows multiple sources to transmit their data simultaneously, i.e., via a non-orthogonal channel. Nazer and Gastpar [7] proposed a PNC method, compute-and-forward (CF) based on nested lattice codes (NLC), for multi-source multi-relay channels. All sources encode their blocks of message into NLC codewords and simultaneously transmit them to the relays. Each relay computes the superimposed codeword signals to obtain the linear combination of the codewords of all sources with a combination integer coefficient vector, then forwards the codeword combination to the destination. The destination can recover the original blocks of all sources from the codeword combinations forwarded from the relays if it receives enough linearly independent (i.e., innovative) codeword combinations. The cooperation between relays can provide the opportunity for the destination to collect enough linearly independent codeword combinations with desired purposes such as obtaining the highest sum rate [8,9] or the highest throughput [10] while the sources also take part.

However, in some cases, codeword combinations are not qualified to be forwarded to the destination, i.e., considered as unsuccessfully received, and the codeword combinations at the different relays might be linearly dependent on each other since all relays compute the superimposed signals independently. Retransmission and feedback sending back to the sources will be needed if the relays cannot provide enough linearly independent codeword combinations to the destination to recover the original blocks of all sources. However, feedback might be lost, has some delay in reaching the sources and causes some energy consumption. These shortcomings are called protocol overhead in this paper.

In the traditional communication network, when a block or a packet is correctly received by the destination, a feedback, i.e., an acknowledgment (ACK), is sent back to the source to manage the next transmission. Negative ACK (NACK) is used to inform about the unsuccessful reception of a sending block. Block ACK (BACK) can be used to reduce the needed ACKs as transmitting a certain number of blocks, and a BACK containing the reception states of the sending blocks is sent back to the source. However, if feedback is lost or its transmission time is significant, (e.g., the transmissions between ground stations and satellite), then the protocol overhead can affect the end-to-end network performance, especially in the lossy multi-hop communication multi-source networks because feedback needs to be forwarded via the intermediate nodes to the sources.

On the other hand, RLNC can be applied to reduce the protocol overhead because an ACK is needed when the receiver can decode the received coded blocks. However, if the number of input blocks is large, then the encoding and decoding computational complexity, which depends on the number of input blocks, especially decoding complexity, will be high and not practical. The large number of blocks is grouped into disjoint generations or chunks [11,12], then the computational complexity can be reduced. Nevertheless, when the number of blocks per chunk, i.e., size of the chunk, is too small, the protocol overhead is still significant. If the message success rate (MSR) of the transmission link is known, then the sources can transmit each chunk with an expected number of coded blocks, and feedback can be avoided [4]. However, if MSR is not constant, then there will be some chunks that are not decodable, i.e., undecodable. To deal with this problem, the works in [13,14] proposed overlapped chunk code (OCC), where a block can belong to more than one chunk. A decoded chunk can be used to help to decode the other undecodable chunks by back-substitution, i.e., blocks from decoded chunks are substituted into the undecodable chunks that also have them as input blocks. The other designs of OCCs and those of the codes similar to OCC were proposed then such as in the works of [15,16,17,18]. These designed codes are mainly for single flow transmission or multicast transmission, i.e., the transmission of a source data. Up to the present, there is no design of OCC for the data transmission in multi-source multi-relay networks.

This paper considers the design and the application of OCC for the data transmissions in multi-source multi-relays networks where CF based on NLC is employed. The designed OCC is denoted as OCC/CF in this paper. The aim is to investigate the advantage of OCC/CF over a feedback-based transmission scheme. In addition, low computational complexity is considered such that the proposed work is applicable to low specification wireless nodes, e.g., wireless sensor nodes. This paper considers varying channel states where only receivers have knowledge of channel coefficients. The blocks of each source message are grouped into chunks. RLNC is done within each chunk before encoding with NLC. Only the transmissions from the sources to the relays are considered. The challenge to apply OCC in a multi-source multi-relay network is how to design OCC/CF such that the decodability of each chunk of all sources at the destination is ensured or the desired network performance is achieved. The contributions of this paper are as follows:

- analyzing the decodability for chunks received at the destination to design OCC for each source and providing a decodability condition to design OCC/CF;
- based on the condition of decodability, designing OCC/CF by employing an OCC with a contiguously overlapping, but non-rounded-end fashion at each source. The design is done by using the empirical rank distribution, i.e., the probability distribution of the number of linearly independent codeword combinations received at the destination per chunk transmission, as in the work of [17,18], and by using the probability distribution of the participation factor of each source to the received codeword combinations per chunk transmission. These two keys depend on the channel states from the sources to the relays, and they are applicable for any channel distribution;
- providing a decoding scheme based on the feature of the employed OCC. The decoding scheme considers the other opportunity of starting decoding besides back-substitution, the combination of chunks. The decoding complexity is bounded by the maximum number of combined chunks, and the storing overhead can be reduced;
- estimating the performance of the designed OCC/CF by following the decoding scheme and using table lookup for all allocations, i.e., the number of innovative blocks per chunk and the number of contiguously overlapped blocks for each source. The estimation is to determine which allocation can provide the desired performance such as high decodability, highest channel efficiency and acceptable decoding complexity;
- reducing the number of candidates for the linear combination coefficient vector computed at each relay. This is achieved by a trade-off between computational latency and the performance in the frame error rate.

The numerical results demonstrate that the design of OCC/CF not only depends on the empirical rank distribution, but also on the probability distribution of the participation factor of each source. The chance to improve the network performance by employing OCC/CF depends on the feedback latency and feedback reception success rate if comparing with a feedback-based CF transmission scheme.

The remainder of this paper is organized as follows. Related works are described in Section 2. A short review of NLC and CF is introduced in Section 3. Section 4 describes the system model of this paper work, which includes the scenario, channel model, encoding scheme at sources and computing at relays, acquiring the linear combination coefficient vector at each, the considered empirical rank distributions and the analysis of decodability. Section 5 talks about the design of OCC/CF by using an OCC and the applied decoding scheme. The estimation of decodability for the OCC/CF designed is given in Section 6. The performance analysis and the reference schemes are described in Section 7. Section 8 shows the numerical results and discussion. At the end, Section 9 gives the conclusion.

## 2. Related Works

To complete the message transmission without the need for feedback, the code to be mentioned would be rateless code where the number of coded blocks is unlimited and the transmitter keeps sending the coded blocks until the receiver can recover all original blocks or packets. Fountain code [19] is an erasure code and a rateless code. The feature of fountain code is low computational complexity in encoding and decoding processes since they are done in the binary field, i.e., $F_{2}$. This includes LTcode [20], Raptor code [21] and online code [22]. The decodability for LT code depends on the degree distribution, which is determined based on the soliton distribution. Degree is the number of input blocks to generate a coded block. The input blocks for each coded block are randomly selected. Raptor code applied the precoding process before encoding such that while a fraction of coded blocks are received, then all original blocks are recoverable. Online codes applied a precoding process for the distributed networks. The decoding process, while employing fountain codes, starts when at least a one-degree coded block, i.e., plain block, exists and stops when there are no more one-degree coded blocks. The decoded blocks are back-substituted into the new received coded blocks, which also have them as input blocks. The application of the inactivation decoding method [23] was studied in the work [24] for the decoding process of LT code and Raptor code to reduce the decoding complexity because the transfer matrix of the received coded blocks, i.e., the coding coefficient matrix of the received coded blocks, is a sparse matrix.

For RLNC, each element of the coding coefficient matrix of the sending coded blocks are randomly drawn from a finite field $F_{q}$ (normally, *q* is enough large, e.g., $q=2^{8}$). The linear independence between coded blocks with RLNC is higher than with sparse network coding (the generated coding coefficient matrix of the coded blocks is a sparse matrix) especially in lossy communication networks, but the computational complexity of RLNC is higher. RLNC was employed within each chunk for OCC proposed in the work of [13] where two overlapping fashion were given: rectangular grid code and diagonal grid code. The number of chunks is finite, but the decodability of received chunks was not clearly analyzed. The overlapping fashion of OCC in the work of [14] is contiguous and in a rounded-end fashion. The decodability is analyzed with chunk size, the number of contiguously overlapped blocks and the number of received coded blocks. However, achieving high decodability, i.e., the probability that a chunk is decodable, requires a large chunk size, which can make the computational complexity more significant. A small sized chunk was analyzed then in their later work [15]. However, the decoding process would start when the receiver has collected a sufficient number of coded blocks of all chunks in the worst case, i.e., when there are no more decodable chunks. Then, higher storing ability at the receiver would be required, and the decoding complexity is still significant. The design of OCC with the other overlapping fashion was proposed in the work of [16], where the overlapped blocks, i.e., the blocks taken from the other chunks, are randomly selected. Although the performance in decodability is better than OCC with the contiguously overlapping fashion [14], the decoding process still might start when a sufficient number coded blocks are received.

Batched sparse (BATs) codes proposed in the work of [17] inherit the feature of rateless code by employing fountain codes as the outer code (chunk size obeys a degree distribution) and random linear network code as the inner code (RLNC is employed within each chunk). The degree distribution is determined using the empirical rank distribution to obtain the optimal performance in achievable rate. The decoding process starts when there is at least a decodable chunk, and back-substitution is done then. The inactivation decoding method might be applied when there are no more decodable chunks. The other design, which also employs the empirical rank distribution, is in the work of [18], where chunk size is fixed. Two degree distributions are defined, and a degree distribution is determined when another degree distribution is fixed to obtain the optimal achievable rate.

This paper provides the design of OCC/CF with a condition of decodability, which can be applied with the designs of codes for single flow transmission, which are described above. This paper employs an OCC in a contiguously overlapping fashion to design OCC/CF because it is simpler to determine which allocation for each source to obtain the desired performance since there are only two variables to be determined for each source. Although its performance in rate (channel efficiency, for this paper) is not higher than the other designs in single flow transmission, it has a potential to reduce the storage overhead and the computational complexity to suit its application with a low specification wireless node in multi-source multi-relay networks.

## 3. Preliminaries

### 3.1. Notation

Boldface letters are used for vectors, e.g., a. The capital boldface letters are for matrices, e.g., G. Superscripts ${}^{T}$ and ${}^{-1}$ refer to matrix transposition operation and inverse operation, respectively. R and Z denote the field of real values and the field of integer values, respectively. In addition, sign · refers to the multiplication operation, and sign × is used to express the size of the matrix.

### 3.2. Nested Lattice Codes

An *n*-dimensional lattice Λ is a linear additive subgroup of $R^{n}$, i.e., if $x_{1},x_{2}\in \Lambda$, then $x_{1}+x_{2}\in \Lambda$ and $-x_{1}\in \Lambda$. A lattice point x∈Λ is generated by the generator matrix $G\in R^{n\times n}$ and an integer vector $b\in Z^{n}$ by:

(1) x=G·b.

The fundamental Voronoi region of Λ, V, is the space that is closer to the origin $x_{o}$$\left(x_{o}=0\right)$ than to the other lattice points. A scaled lattice $\Lambda_{p}=p\cdot \Lambda$ is obtained by scaling the generator matrix of Λ, i.e., $G_{p}=p\cdot G$. A lattice $\Lambda_{p}$ is nested in Λ if $\Lambda_{p}\subseteq \Lambda$. If *p* is a non-zero positive integer, then $\Lambda_{p}=p\cdot \Lambda$ is nested in Λ.

NLC is formed by a coding lattice $\Lambda_{c}$ and a shaping lattice $\Lambda_{s}$, where $\Lambda_{s}\subseteq \Lambda_{c}$. The codebook of NLC is the coset leaders of $\Lambda_{c}/\Lambda_{s}$, i.e., the lattice points (codewords) of $\Lambda_{c}$ that are inside the fundamental Voronoi region of $\Lambda_{s}$, $V_{s}$. If taking $\Lambda_{s}=q\Lambda_{c}$, where *q* is a prime number, and the generator matrix of $\Lambda_{c}$, $G_{c}$, is full rank, then the coding rate of NLC is $R=\log_{2}q$. The number of codewords is $q^{n}$. The feature of NLC is that the linear combination of two codewords is still a codeword. The encoding process of NLC can be done as below.

(2) $$x=\left[G_{c}\cdot b\right]\;\mathrm{mod}\;V_{c},$$

where $b\in F_{q}^{n}$ is the information, **x** is the NLC codeword corresponding to **b** and [ ] mod $V_{s}$ is the operation mapping a lattice point of $\Lambda_{c}$ into $V_{s}$ This operation restricts the transmit power of a sending codeword by an assigned maximum transmit power *P*_max_.

The decoding process can be done as below:

(3) $$b=[G_{c}^{-1}\cdot x]\;\mathrm{mod}\;q,$$

where [ ] mod *q* is the modulo operation by *q* or the operation mapping an integer value into the finite field $F_{q}$.

### 3.3. Compute-and-Forward

If *K* sources transmit their codeword simultaneously, the accumulative codeword at relay *l* can be expressed by:

(4) $$y_{l}=\sum_{k=1}^{K}h_{kl}\cdot x_{k}+z_{l},$$

where $x_{k}\in R^{n}$ is an *n*-dimensional NLC codeword, which is transmitted from source *k* for k∈{1,2,⋯,K}. $h_{kl}\in R$ is a real channel coefficient of the link from source *k* to relay *l*. For the case of a complex channel coefficient, the derivation can be done as in the work of [7]. On the other hand, $z_{l}\in R^{n}$ is additive white Gaussian noise (AWGN).

Relay *l* computes $y_{l}$ to obtain a linear combination of the codewords of *K* sources, $v_{l}$, where:

(5) $$v_{l}=\sum_{k=1}^{K}a_{kl}\cdot x_{k},$$

where $a_{l}={\left[a_{1l},a_{2l},\cdots ,a_{Kl}\right]}^{T}\in Z^{K}$ is a linear combination integer coefficient vector used at relay *l* and $a_{kl}$ is called the *k*-th element of $a_{l}$. $v_{l}$ is mapped into $V_{s}$ to obtain $u_{l}$ before forwarding to obey the transmit power constraint, i.e., $\frac{1}{n}\sum_{n^{\prime }=1}^{n}u_{ln^{\prime }}^{2}\leq P_{\max}$, where $u_{ln^{\prime }}$ is the $n^{\prime }$-th element of $u_{l}$, and $P_{\max}>0$.

(6) $$u_{l}=\left[v_{l}\right]\;\mathrm{mod}\;V_{s}=\left[\sum_{k=1}^{K}\beta_{kl}\cdot x_{k}\right]\;\mathrm{mod}\;V_{s},$$

where $\beta_{l}={\left[\beta_{1l},\beta_{2l},\cdots ,\beta_{kl}\right]}^{T}\in F_{q}^{K}$ and $\beta_{l}=\left[a_{l}\right]$ mod *q*. ***β****_l_* is a combination coefficient vector for codeword combination **u**_l_.

## 4. System Model

### 4.1. Scenario

**Figure 1:**
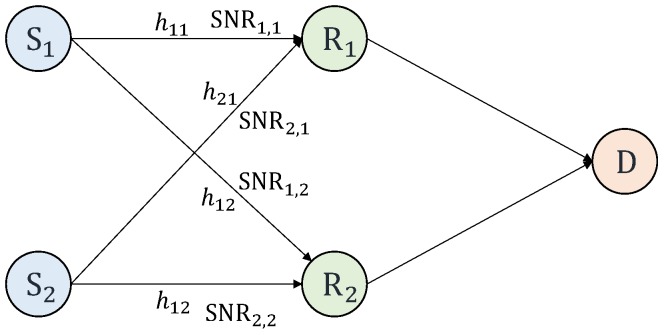
Scenario for the case of a two-source two-relay single-destination network. *h_kl_* is the channel coefficient corresponding to the instantaneous received signal-to-noise ratio (SNR) of the link from source *k* to relay *l*, where *k* ∈ {1,2, …, *K*} and *l* ∈ {1,2, …, *L*}. SNR_*kl*_ denotes the average received SNR of the link from source *k* to relay *l*.

This paper takes a scenario of a *K* sources *L* relays single-destination network as shown in Figure 1, which is in the case *K* = *L* = 2. Each node is equipped with a single antenna. The direct links from the sources to the destination are not considered, and only the transmissions from the sources to the relays are considered. In this paper, all sources apply the NLC with the same coding rate *R* = log_2_ *q*. This paper assumes that the transmissions from the relays to the destination are lossless. The process of forwarding the codeword combinations to the destination can be done as in the works of [8,9] by exploiting the coordination from the destination via control messages between the relays and the destination to select which codeword combinations are to be forwarded and which relays to forward.

This scenario is considered as data collection in wireless sensor networks or data backhauling in ultra-dense networks, and it is a part of the topologies of these networks. On the other hand, if its application in cognitive radio (CR) network is considered, the primary user (PU) is one of *K* sources, and the other sources are secondary users (SUs). Alternatively, all sources can be assigned as SUs. There is a relay assigned for each source if the transmissions (used for reference schemes) via the orthogonal channel are considered.

### 4.2. Channel Model

This paper assumes that time is slotted and synchronized. Only real channel coefficients are considered, and the block channel fading is assumed, i.e., the channel coefficient for a whole block signal within a time slot along a channel link is constant. In addition, Rayleigh fading is considered, and the channel coefficient is independently and identically distributed for each channel link. Hence, the real channel coefficient is normally distributed. The average received signal-to-noise ratio for the link from source *k* to relay *l* is denoted by ${\mathrm{SNR}}_{kl}$. On the other hand, AWGN has zero mean and unit variance in this paper.

### 4.3. Computing Combination Coefficient Vector

$R_{l}\left(h_{l},a_{l}\right)$ is defined as the computation rate region corresponding to the channel coefficient vector $h_{l}={\left[h_{1l},h_{2l},\cdots ,h_{Kl}\right]}^{T}$ and correspondent $a_{l}$. According to the work in [7], $R_{l}$ is achievable for any large enough *n* and for the existing encoders and decoders such that the receiver can recover the desired codeword combination with $a_{l}\neq 0$ with the average probability of error ϵ>0 if the maximum coding rate of all sources, i.e., *R* for this paper, satisfies the condition:

(7) $$R_{l}\left(h_{l},a_{l}\right)>R.$$

In this paper, $a_{l}$ is determined by applying the method proposed by U. Fincke and M. Pohst [25] as in the work of [26] to obtain the highest $R_{l}\left(h_{l},a_{l}\right)$. By considering the hardware specification of sensor nodes, this paper exploits Condition (7) to reduce the computational overhead by reducing the number of candidates of $a_{l}$ in searching, for which $a_{l}$ can provide the highest $R_{l}\left(h_{l},a_{l}\right)$. In addition, Condition (7) is also used to filter codeword combination for forwarding to the destination at each relay. Since $R=\log_{2}q$, the higher value *q* results in a high message loss rate. In this paper, only a small value of *q* is considered. Reducing the number of candidates of $a_{l}$, i.e., reducing the bounds of the value of the elements of $a_{l}$, can be done as in the works of [26,27] by replacing the condition $R_{l}\left(h_{l},a_{l}\right)>0$ with $R_{l}\left(h_{l},a_{l}\right)>R$.

**Figure 2:**
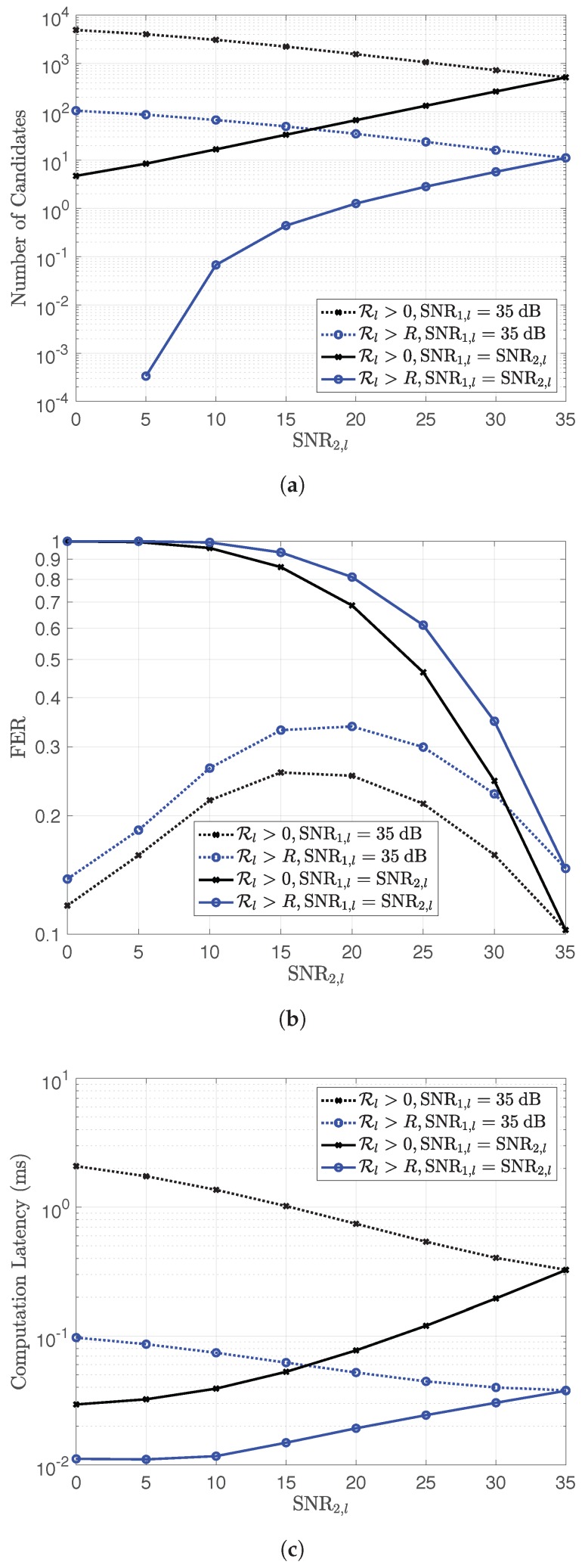
The performance applying condition $R_{l}(h_{l},a_{l})>R$ compared with condition $R_{l}(h_{l},a_{l})>0$. (**a**) Number of candidates; (**b**) frame error rate (FER); (**c**) computational latency.

However, this modification causes some decrease in performance in the block error rate or frame error rate (FER) because codeword combination might be correctly received without satisfying Condition (7). By comparing with the case that applies condition $R_{l}\left(h_{l},a_{l}\right)>0$, the number of candidates, FER and computational latency are shown in Figure 2. The specification of the employed platform is shown in Table 1. The lattice code $E_{8}/7E_{8}$ is used for NLC in this comparison, where q=7, and $E_{8}$ is a well-known n=8 lattice.

The result is obtained by considering the codeword combinations of two sources at relay *l* and taking ${\mathrm{SNR}}_{2,1}=0\to 35\;\mathrm{dB}$ and ${\mathrm{SNR}}_{1,l}$ with two cases: ${\mathrm{SNR}}_{1,l}=35\;\mathrm{dB}$ and ${\mathrm{SNR}}_{1,l}={\mathrm{SNR}}_{2,l}=0\to 35\;\mathrm{dB}$. The FER for condition $R_{l}\left(h_{l},a_{l}\right)>0$ was obtained by comparing the codeword combination with the combination of the original codewords. For the case with Condition (7), the codeword combination is filtered with Condition (7) first before comparing with the combination of the original codewords. From Figure 2, this setting performs the trade-off between the computational latency and the FER performance.

### 4.4. Encoding and Computing

A big file message is divided into small blocks, and blocks are selected to group into chunks or batches. For source *k* where k∈{1,2,⋯,K}, the *i*-th chunk consists of $d_{k}^{\left(i\right)}$ blocks and is expressed by $B_{k}^{\left(i\right)}=\left[b_{k1}^{\left(i\right)},b_{k2}^{\left(i\right)},\cdots ,b_{kd_{k}^{\left(i\right)}}^{\left(i\right)}\right]$. $D^{\left(i\right)}$ and $d_{\max}$ denote $\sum_{k=1}^{K}d_{k}^{\left(i\right)}$ and $\max\{d_{k}^{\left(i\right)},1\leq k\leq K,\forall i\}$, respectively. RLNC is applied among chunks to generate *M* coded blocks, $W_{k}^{\left(i\right)}=\left[w_{k1}^{\left(i\right)},w_{k2}^{\left(i\right)},\cdots ,w_{kM}^{\left(i\right)}\right]$. The *m*-th coded block, $w_{km}^{\left(i\right)}$, is obtained by:

(8) $$w_{km}^{\left(i\right)}=\left[\sum_{d=1}^{d_{k}^{\left(i\right)}}\chi_{kmd}^{\left(i\right)}\cdot b_{kd}^{\left(i\right)}\right]\;\mathrm{mod}\;q,$$

where $\chi_{km}^{\left(i\right)}={\left[\chi_{km1}^{\left(i\right)},\chi_{km2}^{\left(i\right)},\cdots ,\chi_{kmd_{k}}^{\left(i\right)}\right]}^{T}$ is randomly drawn from $F_{q}^{d_{k}^{\left(i\right)}}$. It is the coding vector of coded block $w_{km}^{\left(i\right)}$. Superscript ${}^{\left(i\right)}$ is sometimes omitted here for convenience. The computational complexity of the encoding process depends on chunk size $d_{k}^{\left(i\right)}$.

**Figure 3:**
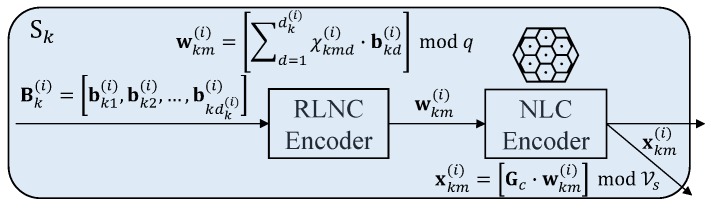
Encoding process at source *k* for chunk *i*. RLNC, random linear network coding.

The coded blocks of each chunk are then NLC encoded before transmitting to generate *M* NLC codewords, $X_{k}^{\left(i\right)}=\left[x_{k1}^{\left(i\right)},x_{k2}^{\left(i\right)},\cdots ,x_{kM}^{\left(i\right)}\right]$, as shown in Figure 3. All sources transmit these *M* codewords for each chunk simultaneously to the relays.

**Figure 4:**
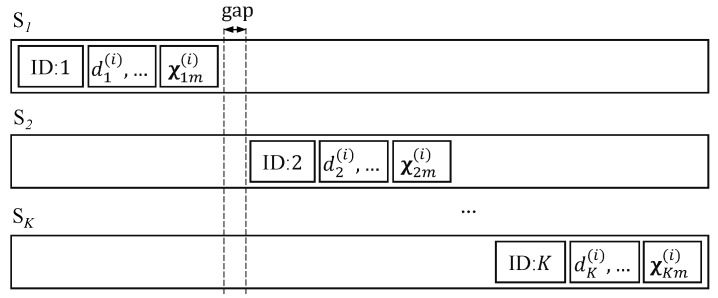
Locations of the attached information in the header of the sending frame of all sources for chunk *i*, where “$d_{k}^{\left(i\right)}$, …” refers to the information about chunk *i* for source *k* such as chunk size $d_{k}^{\left(i\right)}$, information about overlapped blocks, etc.

The coding vector $\chi_{km}^{\left(i\right)}$ and the information of chunk *i* for source *k*, such as source ID (*k*), $d_{k}^{\left(i\right)}$, etc., can be attached to the transmitting data, e.g., at the header of the frame. However, the location of the attached information for a source should not overlap with those of the other sources, as shown in Figure 4. Hence, the small chunk size is preferred for the header with a limited length. This paper assumes that the length of the attached information is negligible compared with the length of the sending block. Alternatively, this information can be known by the receivers (relays or destination) by broadcasting from each source, for example. This paper assumes that the content of this information is correctly received.

Relay *l* for l∈{1,2,⋯,L} computes the superposition of *K* codewords $\left[x_{1m}^{\left(i\right)},x_{2m}^{\left(i\right)},\cdots ,x_{Km}^{\left(i\right)}\right]$ to obtain their linear combination $u_{lm}^{\left(i\right)}\in R^{n}$ to forward to the destination, which is:

(9) $$u_{lm}^{\left(i\right)}=\left[\sum_{k=1}^{K}\beta_{klm}^{\left(i\right)}\cdot x_{km}^{\left(i\right)}\right]\;\mathrm{mod}\;V_{s},$$

where $\beta_{lm}^{\left(i\right)}={\left[\beta_{1lm}^{\left(i\right)},\beta_{2lm}^{\left(i\right)},\cdots ,\beta_{klm}^{\left(i\right)}\right]}^{T}\in F_{q}^{K}$ s the combination coefficient vector computed at relay *l* for the *m*-th blocks of all sources for chunk *i*. Then, the combined coding coefficient vector of $u_{lm}^{\left(i\right)},c_{lm}^{\left(i\right)}\in F_{q}^{D}$, is:

(10) $$c_{lm}^{\left(i\right)}=({\left[\beta_{1lm}^{\left(i\right)}\cdot \chi_{1m}^{\left(i\right)},\beta_{2lm}^{\left(i\right)}\cdot \chi_{2m}^{\left(i\right)},\cdots ,\beta_{Klm}^{\left(i\right)}\cdot \chi_{Km}^{\left(i\right)}\right]}^{T})\;\mathrm{mod}\;q.$$

### 4.5. Empirical Probability Distributions

Since lossless transmissions from the relays to the destination are assumed, the total number of linearly independent codeword combinations at the relays for each chunk is the same at the destination. $r^{\left(i\right)}$ (*r* for any chunk) denotes the number of linearly independent codeword combinations correctly received at the relays (destination) for chunk *i*. Hence, $r^{\left(i\right)}$ is the rank of matrix $C^{\left(i\right)}\in F_{q}^{D^{\left(i\right)}\times r^{\left(i\right)}}$, which is a set of $r^{\left(i\right)}$ linearly independent vectors taken from L·M vectors $\left[c_{11}^{\left(i\right)},\cdots ,c_{L1}^{\left(i\right)},c_{12}^{\left(i\right)},\cdots ,c_{L2}^{\left(i\right)},\cdots ,c_{LM}^{\left(i\right)}\right]$.

The original blocks of all sources for chunk *i* are recoverable if there are $D^{\left(i\right)}$ linearly independent received codeword combinations for chunk *i*, i.e., $r^{\left(i\right)}=D^{\left(i\right)}$. If the channel state is stable, i.e., $r^{\left(i\right)}$ is constant for all *i*, all chunks can be decoded with a suitable value of *M* without the need for feedback from the destination. However, with the unstable channel state, $r^{\left(i\right)}$ varies with different chunks. Hence, without the aid of feedback, there are some chunks that are undecodable. As in the works of [17,28], in this paper, $\rho \left(r\right)$ denotes the empirical probability distribution of *r*, for $r\in \{0,1,\cdots ,D_{\max}\}$, where $D_{\max}=\max\left\{D^{\left(i\right)},\forall i\right\}$.

On the other hand, in this paper, $\theta_{k}^{\left(i\right)}$ ($\theta_{k}$ for any chunk) denotes the rank of the part of the matrix $C^{\left(i\right)}$ from row $1+\sum_{k^{\prime }=1}^{k-1}d_{k^{\prime }}^{\left(i\right)}$ to row $\sum_{k^{\prime }=1}^{k}d_{k^{\prime }}^{\left(i\right)}$. $\theta_{k}^{\left(i\right)}$ is defined as the participation factor of source *k* in $C^{\left(i\right)}$, i.e., in the forwarded codeword combinations of chunk *i*. In addition, $\lambda_{k}\left(\theta_{k}\right)$ denotes the empirical probability distribution of $\theta_{k}$, for $\theta_{k}\in \left\{0,1,\cdots ,d_{k}\right\}$.

In practical applications, $\rho \left(r\right)$ and $\lambda_{k}\left(\theta_{k}\right)$ can be collected by employing a feedback-based transmission scheme only for the chunks without feedback loss, as in the work of [29], for example. The overhead caused by the linear dependence between coded blocks and between codeword combinations, i.e., due to the small value of finite field size *q*, is taken into account in the data collections of $\rho \left(r\right)$ and $\lambda_{k}\left(\theta_{k}\right)$. In addition, exploiting the probability distributions for the design of OCC/CF enables OCC/CF to be applicable to the other channel distributions, ensuring its robustness.

### 4.6. Decodability

In order to analyze the decodability, in this paper, $p_{d}$ and $p_{k}$ denote the probabilities that chunk *i* is decodable, i.e., $r^{\left(i\right)}=D^{\left(i\right)}$ and $\theta_{k}^{\left(i\right)}=d_{k}^{\left(i\right)}$, respectively, when employing an OCC, which is designed by using $\rho \left(r\right)$ and $\lambda_{k}\left(\theta_{k}\right)$, respectively, in single-transmission flow, i.e., transmission from a source to a relay via an orthogonal channel. The overlapping fashions of OCCs corresponding to $\rho \left(r\right)$ and $\lambda_{k}\left(\theta_{k}\right)$ are the same.

This paper considers the case that K≥L, $M=d_{\max}$, and source *k* generates *M* coded blocks by the RLNC encoder with $d_{k}^{\left(i\right)}$ linearly independent coded blocks for chunk *i* and all *k*, i.e., $\left[\chi_{k1}^{\left(i\right)},\chi_{k2}^{\left(i\right)},\cdots ,\chi_{kM}^{\left(i\right)}\right]\in F_{q}^{d_{k}^{\left(i\right)}\times M}$ would be pseudorandom to ensure the linear independence between coded blocks. When employing OCC/CF, the codeword combinations of chunk *i* are recoverable at the destination if there are $D^{\left(i\right)}$ received linearly independent codeword combinations, i.e., $r^{\left(i\right)}=D^{\left(i\right)}$. To determine the probability that a chunk is decodable, this paper studies two cases as below:

- Case I: $\beta_{lm}^{\left(i\right)}=\left[\beta_{1lm}^{\left(i\right)},\beta_{2lm}^{\left(i\right)},\cdots ,\beta_{Klm}^{\left(i\right)}\right]$ is a unit vector, i.e., only an element of $\beta_{lm}^{\left(i\right)}$ is equal to one, and the others are zero;
- Case II: $\beta_{lm}^{\left(i\right)}$ does not have zero elements; there are only *M* linearly independent codeword combinations, and they are only forwarded by a relay.

For Case I, $r^{\left(i\right)}$ can be written as $r^{\left(i\right)}=\sum_{k=1}^{K}\theta_{k}^{\left(i\right)}$. Hence, the decodability of each chunk only depends on the OCC design using $\lambda_{k}\left(\theta_{k}\right)$ for all *k*. In this case, the original blocks of each source can be recovered independently since every received codeword combination corresponds to the coded blocks from only one source. By assuming that chunk *i* for all sources is decodable, i.e., $r^{\left(i\right)}=D^{\left(i\right)}$, if the chunks of all sources are decodable, thus the probability that a chunk for all sources is decodable, $p_{\mathrm{dec}}$, can be written as $p_{\mathrm{dec}}=\prod_{k=1}^{K}p_{k}$, which is independent of $p_{d}$ or $\rho \left(r\right)$.

For Case II, since $M\geq \max\{d_{k}^{\left(i\right)},1\leq k\leq K,\forall i\}$ and there are $d_{k}^{\left(i\right)}$ linearly independent coded blocks from source *k* for chunk *i*, hence $\theta_{k}^{\left(i\right)}=d_{k}^{\left(i\right)}$ for chunk *i*. This case assumes that chunk *i* is not decodable and there are $\gamma_{k}^{\left(i\right)}\leq d_{k}^{\left(i\right)}$ blocks inside chunk *i* for source *k* with k∈{1,2,⋯,K}, which also belong to the other chunks. If these $\gamma_{k}^{\left(i\right)}$ blocks have been already recovered with the decoded chunks, then there are still $\sum_{k=1}^{K}\left(d_{k}^{\left(i\right)}-\gamma_{k}^{\left(i\right)}\right)=D^{\left(i\right)}-\sum_{k=1}^{K}\gamma_{k}^{\left(i\right)}$ to recover for chunk *i*. From another point of view, it is equivalent to the case that matrix $C^{\left(i\right)}$ has $\gamma_{k}^{\left(i\right)}$ eliminated rows, which are between row $1+\sum_{k^{\prime }=1}^{k-1}d_{k^{\prime }}^{\left(i\right)}$ and row $\sum_{k^{\prime }=1}^{k}d_{k^{\prime }}^{\left(i\right)}$, and becomes a $\left(D^{\left(i\right)}-\sum_{k=1}^{K}\gamma_{k}^{\left(i\right)}\right)\times M$ matrix, $C^{\prime (i)}$. Since $\chi_{km}^{\left(i\right)}$ is randomly drawn from $F_{q}^{d_{k}^{\left(i\right)}}$ for m∈{1,2,⋯,M}, hence $C^{\prime (i)}$ can be approximately also drawn from $F_{q}^{d_{k}^{\left(i\right)}}$. In addition, $C^{\prime (i)}$ can be approximately obtained by eliminating $\sum_{k=1}^{K}\gamma_{k}^{\left(i\right)}$ rows from a $D^{\left(i\right)}\times M$ matrix, which is randomly drawn from $F_{q}^{D^{\left(i\right)}\times M}$. It looks like $\sum_{k=1}^{K}\gamma_{k}^{\left(i\right)}$ blocks are back-substituted into a chunk *i* when employing OCC in single-flow transmission. Then, the decodability of each chunk when employing OCC/CF is the same as when employing OCC designed using $\rho \left(r\right)$ in single-flow transmission. Hence, in this case, $p_{\mathrm{dec}}=p_{d}$. With Case II, the feature is that already recovered $\gamma_{k}^{\left(i\right)}\leq d_{k}^{\left(i\right)}$ blocks can be back-substituted into chunk *i* without waste.

**Figure 5:**
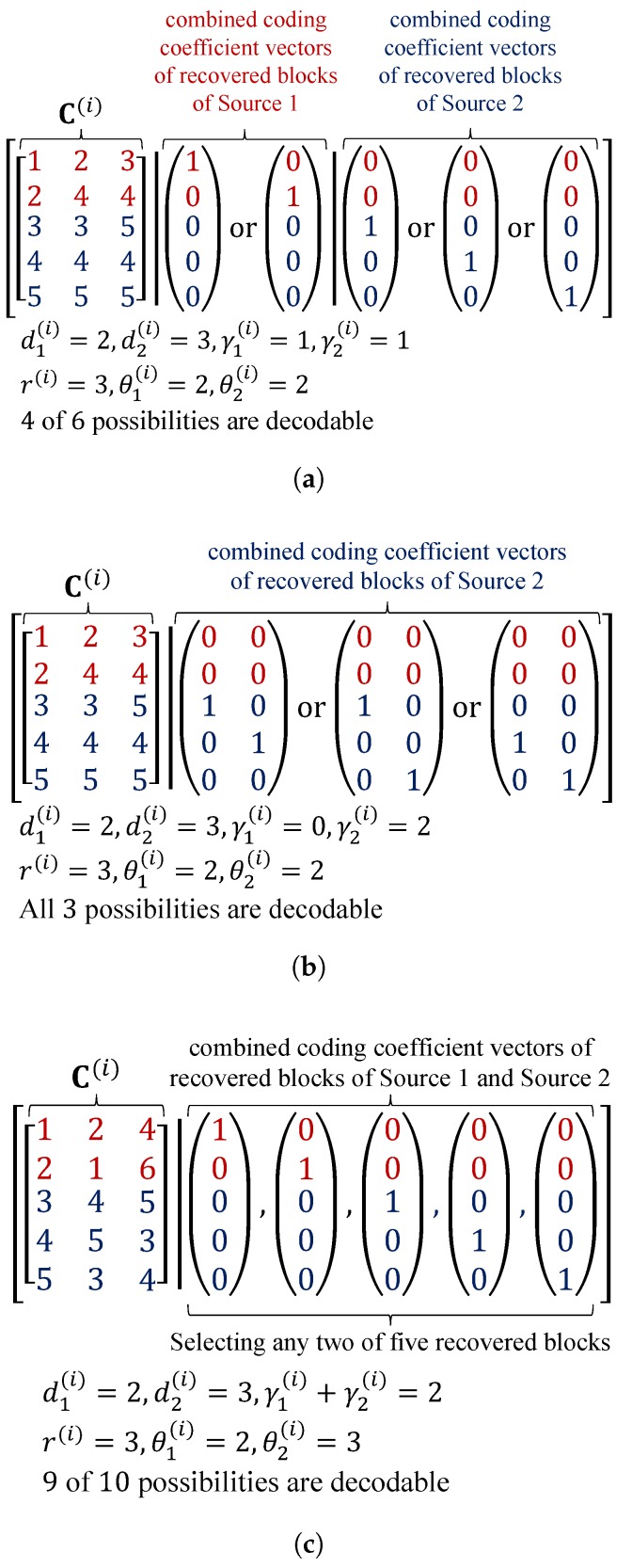
Example from point of view of the combined coding coefficient matrix. (**a**) $\gamma_{1}^{\left(i\right)}=1,\gamma_{2}^{\left(i\right)}=1,\theta_{1}^{\left(i\right)}=2,\theta_{2}^{\left(i\right)}=2$; (**b**) $\gamma_{1}^{\left(i\right)}=0,\gamma_{2}^{\left(i\right)}=2,\theta_{1}^{\left(i\right)}=2,\theta_{2}^{\left(i\right)}=2$; (**c**) $\gamma_{1}^{\left(i\right)}+\gamma_{2}^{\left(i\right)}=2,\theta_{1}^{\left(i\right)}=2,\theta_{2}^{\left(i\right)}=3$. The decodability of chunk *i* is given with different possibilities of recovered blocks.

In contrast, for the other case, by taking $\theta_{k}^{\left(i\right)}<d_{k}^{\left(i\right)}$ and $\gamma_{k}^{\left(i\right)}=d_{k}^{\left(i\right)}-\theta_{k}^{\left(i\right)}$ for example, these recovered blocks can successfully increase the number of linearly independent received coded blocks in chunk *i* if they are linearly independent of the existing received coded blocks in chunk *i*. In addition, the value of $\gamma_{k}^{\left(i\right)}$ should be appropriately selected by using $\theta_{k}^{\left(i\right)}$ or $\lambda_{k}\left(\theta_{k}\right)$ for all chunks. From the point of view of $C^{\left(i\right)}$, an example taking K=2, $d_{1}^{\left(i\right)}=2$, $d_{1}^{\left(i\right)}=2$, M=3, $\gamma_{1}^{\left(i\right)}+\gamma_{2}^{\left(i\right)}=2$ (according to the OCC design using $\rho \left(r\right)$) and q=7 is shown in Figure 5. In Figure 5a,b, $r^{\left(i\right)}=3$, $\theta_{1}^{\left(i\right)}=2$ and $\theta_{2}^{\left(i\right)}=2$ are given. By taking $\gamma_{1}^{\left(i\right)}=1$ and $\gamma_{2}^{\left(i\right)}=1$, then there are five linearly independent blocks in chunk *i* after back-substitution, i.e., chunk *i* is decodable, with four out of six chances. On the other hand, if taking $\gamma_{1}^{\left(i\right)}=0$, $\gamma_{2}^{\left(i\right)}=2$ as in Figure 5b, then chunk *i* is decodable with all three possibilities. Therefore, a suitable selection of $\gamma_{1}^{\left(i\right)}$ and $\gamma_{2}^{\left(i\right)}$ can provide better performance for OCC/CF. Hence, the OCC design using $\lambda_{k}\left(\theta_{k}\right)$ for all *k* is needed. In Figure 5c, $r^{\left(i\right)}=3$, $\theta_{1}^{\left(i\right)}=2$ and $\theta_{2}^{\left(i\right)}=3$ are given. By taking any two different recovered blocks, chunk *i* is decodable with nine of ten chances. The undecodable outcome should be caused by the selection of $\gamma_{1}^{\left(i\right)}+\gamma_{2}^{\left(i\right)}=2$, i.e., the OCC design using $\rho \left(r\right)$. Figure 5c represents Case II where $\theta_{1}^{\left(i\right)}=d_{1}^{\left(i\right)}=2$, $\theta_{2}^{\left(i\right)}=d_{2}^{\left(i\right)}=3$.

For the general case, by combining the two cases above, the effective probability that each chunk is decodable when OCC/CF is applied, denoted by $p_{d\mathrm{eff}}$, can be approximately obtained by:

(11) $$p_{d\mathrm{eff}}=p_{d}\cdot \prod_{k=1}^{K}p_{k}.$$

On the other hand, for the case that K>L>1, the values of *M* and $d_{k}^{\left(i\right)}$ for k∈{1,2,⋯,K} should be selected appropriately such that any chunk *i* can be decoded by itself, i.e., $r^{\left(i\right)}=D^{\left(i\right)}$. For example, if taking $d_{1}^{\left(i\right)}=d_{2}^{\left(i\right)}=\cdots =d_{K}^{\left(i\right)}=d_{\max}$ for all *i*, then $d_{\max}$ should be chosen as a multiple of *L*, and $M\geq \frac{K\cdot d_{\max}}{L}$ to ensure that there is at most L·M codeword combinations to recover $K\cdot d_{\max}$ original blocks. For the case that 1<K≤L, *M* can be taken by $M\geq d_{\max}$ for any value of $d_{k}^{\left(i\right)}>0$, ∀k.

### 4.7. Channel Efficiency

In this paper, channel efficiency is defined as the ratio of the total number of decoded blocks from all sources to the total transmission time (the total number of time slots for OCC/CF or for the transmission schemes without the need for feedback from the relays) taken from the sources to the relays. η and $\eta_{\mathrm{eff}}$ denote the channel efficiencies corresponding to $p_{d}$ and $p_{d\mathrm{eff}}$, respectively.

For a *K*-source *L*-relay network, the ideal value of channel efficiency, which is obtained with lossless transmission and without linear dependence between codeword combinations, is min{K,L}. Thus, for L=1, the channel efficiency would be like in the case of single flow transmission via an orthogonal channel. Hence, applying an orthogonal channel might be a better option. This paper only considers the case that L>1.

On the other hand, $\bar{\rho }$ denotes $\sum_{r=1}^{D_{\max}}r\cdot \rho \left(r\right)$, and $\bar{\eta }$ denotes $\frac{\bar{\rho }}{M}$. $\bar{\eta }$ is called channel capacity in this paper, i.e., the upper bound of $\eta_{\mathrm{eff}}$. Many OCC designs in single-flow transmission try to obtain $\eta_{\mathrm{eff}}$ close to $\bar{\eta }$. In this paper, the (design) overhead is defined as the gap between $\eta_{\mathrm{eff}}$ and $\bar{\eta }$.

## 5. Design with an Overlapped Chunked Code

### 5.1. Encoding

**Figure 6:**
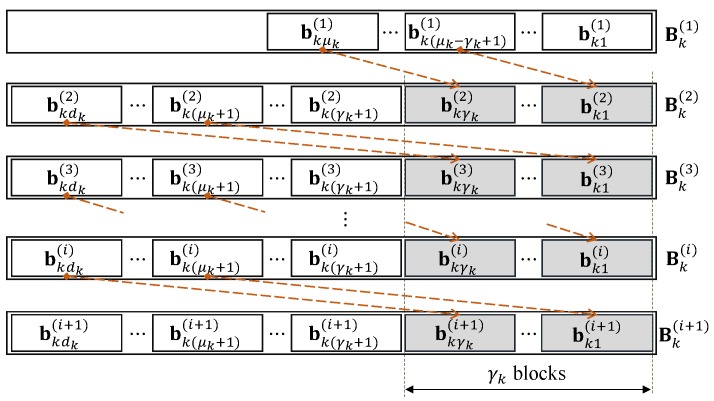
Applied overlapping fashion of overlapped chunked codes (OCC) for source *k*. The blocks in grey for chunk *i* are the blocks taken from the previous chunk, i.e., chunk i−1.

This paper applies an OCC in a contiguously overlapping fashion, which is similar to the works of [14,15], but not in a rounded-end fashion for the design of OCC/CF in a multi-source multi-relay network where CF based on NLC is employed. The applied overlapping fashion is shown in Figure 6.

In this fashion, for source *k* and each chunk, there are $\mu_{k}>0$ innovative blocks, i.e., linearly independent blocks if comparing to the blocks of the other chunks, and there are $\gamma_{k}$ overlapped blocks between two contiguous chunks. Hence, there are $d_{k}=\mu_{k}+\gamma_{k}$ blocks for all chunks except the first chunk where there are only $\mu_{k}$ blocks, i.e., $d_{k}^{\left(1\right)}=\mu_{k}$, since it is not the rounded-end fashion. μ and γ are defined as $\sum_{k=1}^{K}\mu_{k}$ and $\sum_{k=1}^{K}\gamma_{k}$, respectively. There are $\min\{M,d_{k}^{\left(i\right)}\}$ linearly independent coded blocks among *M* coded blocks for chunk *i* and source *k*, where $\left[\chi_{k1}^{\left(i\right)},\chi_{k2}^{\left(i\right)},\cdots ,\chi_{kM}^{\left(i\right)}\right]\in F_{q}^{d_{k}^{\left(i\right)}\times M}$ should be pseudorandomly generated to achieve this goal.

### 5.2. Decoding

The feature of OCC is that a decoded chunk can help the other undecodable chunks in decoding by using back-substitution (b.s). The recovered blocks of the decoded chunk are substituted into the undecodable chunks that consist of the same blocks, i.e., the overlapped blocks. Thus, the number of linearly independent codeword combinations of the back-substituted chunks might be increased, and it depends on the value of *q* and the pairs $\left(d_{k},\gamma_{k}\right)$ for all *k* [28]. With the OCC employed in this paper, for chunk *i*, left back substitution (l.b.s) and right back-substitution (r.b.s) denote b.s by the decoded neighboring chunk on the left, i.e., chunk i−1, and on the right, i.e., chunk i+1, respectively.

**Figure 7:**
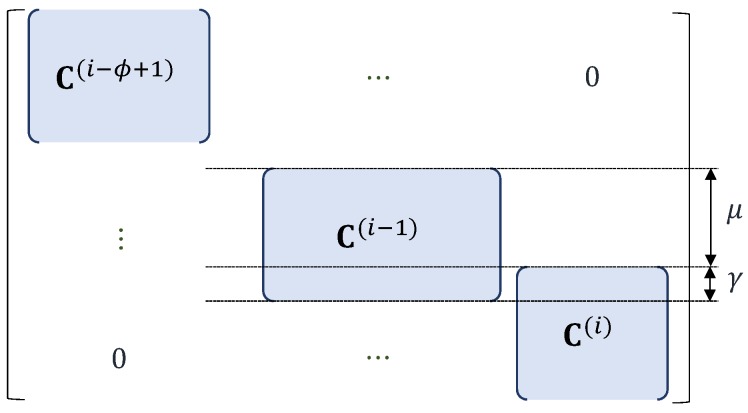
The form of combined coding coefficient matrix of the chain of chunks with length ϕ.

In addition to b.s, this paper considers the other decoding opportunity, called combination of chunks (co.cs) for the applied OCC. co.cs combines the contiguous undecodable chunks into the form of chain of chunks (ch.cs) with length ϕ≥1, where ϕ is the number of the combined chunks. The decoding process can start without the need for at least an already decoded neighboring chunk as with b.s. The form of combined coding coefficient matrix of co.cs, $C_{c}$, is shown in Figure 7.

A ch.cs is decodable if the rank of $C_{c}$, $\mathrm{rank}\left(C_{c}\right)$, is equal to the total number of original blocks inside that ch.cs, which is denoted by $r_{\mathrm{ch}}$. A ch.cs is considered as a directly undecodable ch.cs without waiting to receive a new chunk if $\mathrm{rank}\left(C_{c}\right)$ is lower than a threshold value denoted by $r_{\mathrm{th}}$. $r_{\mathrm{co}}$ and $r_{\mathrm{th}}$ are determined as described in Algorithm 1 by using the feature of the applied OCC. Chunk *i* can participate in the co.cs process if $r_{p}^{\left(i\right)}\leq r^{\left(i\right)}\leq D^{\left(i\right)}-1$, where $r_{p}^{\left(i\right)}$ is determined as described in Algorithm 2. In Algorithms 1 and 2, l.b.s.s$\left(i\right)$ and r.b.s.s$\left(i\right)$ refer to the state of l.b.s and r.b.s, respectively, for considered chunk *i*. l.b.s.s$\left(i\right)$ and r.b.s.s$\left(i\right)$ declare whether undecodable chunk *i* has not been back-substituted by its left neighboring decoded chunk, i.e., chunk i−1, and by its right neighboring decoded chunk, i.e., chunk i+1, respectively. $r_{p}^{\left(t\right)}=0$ means that chunk *t* cannot participate in co.cs, and its decodability depends on $r^{\left(t\right)}$. The process of co.cs is described in Algorithm 3, where d.s(ch.cs) refers to the decodability state of the currently obtained ch.cs.

The decoding process can be done as described in Algorithm 4, where d.s$\left(t\right)$ and d.s$\left(t-\phi +1:t\right)$ declare whether chunk *t* and ch.cs, combining from chunk *t* back to t−ϕ+1, respectively, are decoded or not. The decoding process for a ch.cs with length ϕ≥2 can be done by using the inactivation decoding method [23] in order to reduce the decoding complexity. However, Gaussian elimination is applied for the decoding process in this paper.

The chunks that are considered as directly undecodable chunks without waiting for the next received chunks can become decodable by the aid of feedback from the destination back to the sources. Otherwise, they can be discarded in order to reduce the storage overhead if they do not affect the recovery of all blocks, i.e., the original message. The latter option can be achievable by applying precoding before OCC at each source. With precoding, the original blocks can be recovered when a fraction of all coded blocks is decoded [12].

**Algorithm 1** Determining $r_{\mathrm{th}}$ and $r_{\mathrm{ch}}$ of a co.cs starting from chunk *t* with length ϕ.
1:
**if** t−ϕ+1=1 **then**
2: $r_{\mathrm{ch}}=\phi \cdot \mu$3: **if** r.b.s.s$\left(t\right)=$ **true then**4:  $r_{\mathrm{th}}=\phi \cdot \mu$5: **else**6:  $r_{\mathrm{th}}=\phi \cdot \mu -\gamma$7: **end if**8:
**else**
9: $r_{\mathrm{ch}}=\phi \cdot \mu +\gamma$10: **if** l.b.s.s$\left(t-\phi +1\right)=$**true and** r.b.s.s$\left(t\right)=$ **true then**11:  $r_{\mathrm{th}}=\phi \cdot \mu +\gamma$12: **else if** l.b.s.s$\left(t-\phi +1\right)=$ **true and** r.b.s.s$\left(t\right)=$ **false then**13:  $r_{\mathrm{th}}=\phi \cdot \mu$14: **else if** l.b.s.s$\left(t-\phi +1\right)=$ **false and** r.b.s.s$\left(t\right)=$ **true then**15:  $r_{\mathrm{th}}=\phi \cdot \mu$16: **else if** l.b.s.s$\left(t-\phi +1\right)=$ **false and** r.b.s.s$\left(t\right)=$ **false then**17:  $r_{\mathrm{th}}=\phi \cdot \mu -\gamma$18: **end if**19:
**end if**



**Algorithm 2** Determining $r_{p}^{\left(t\right)}$ for chunk *t*.
1:
**if** t=1 **then**
2: **if** r.b.s.s$\left(t\right)=$ **true then**3:  $r_{p}^{\left(t\right)}=0$4: **else**5:  $r_{p}^{\left(t\right)}=\mu -\gamma +1$6: **end if**7:
**else**
8: **if** l.b.s.s$\left(t\right)=$ **true and** r.b.s.s$\left(t\right)=$ **true then**9:  $r_{p}^{\left(t\right)}=0$10: **else if** l.b.s.s$\left(t\right)=$ **true and** r.b.s.s$\left(t\right)=$ **false then**11:  $r_{p}^{\left(t\right)}=\mu +1$12: **else if** l.b.s.s$\left(t\right)=$ **false and** r.b.s.s$\left(t\right)=$ **true then**13:  $r_{p}^{\left(t\right)}=\mu +1$14: **else if** l.b.s.s$\left(t\right)=$ **false and** r.b.s.s$\left(t\right)=$ **false then**15:  $r_{p}^{\left(t\right)}=\mu -\gamma +2$16: **end if**17:
**end if**



**Algorithm 3** Combination of chunks.
1:Starting from chunk *i*Taking t=i, d.s(ch.cs)= **false**, ϕ=1, $C_{c}=C^{\left(t\right)}$2:
**if** $\mathrm{rank}\left(C_{c}\right)=r_{\mathrm{ch}}$ **then**
3: Update d.s(ch.cs)= **true**4: **return** d.s(ch.cs),ϕ5:
**else**
6: **if** t=1 **or** $\mathrm{rank}\left(C_{c}\right)<r_{p}^{\left(t\right)}$ **or** $l.b.s.s\left(t\right)=$ **true then**7:  **return** d.s(ch.cs),ϕ8: **end if**9:
**end if**
10:
**while** t−1>0 **and** $r_{p}^{\left(t-1\right)}\leq r^{\left(t-1\right)}\leq D-1$ **and** $r.b.s.s\left(t-1\right)=$ **false do**
11: Update ϕ=ϕ+1, $C_{c}=\left[C_{c},C^{\left(t-1\right)}\right]$12: **if** $\mathrm{rank}\left(C_{c}\right)=r_{\mathrm{ch}}$ **then**13:  Update d.s(ch.cs)= **true**14:  **return** d.s(ch.cs),ϕ15: **else**16:  Update t=t−117: **end if**18:
**end while**



**Algorithm 4** Decoding process.
1:Obtaining the codeword combinations of chunk *i*2:Take t=i3:
**if** $r^{\left(t\right)}=D$ **and** d.s(t)= **false then**
4: Conduct decoding and update d.s(t)= **true**, t=t−15: **Go to** 36:
**else**
7: **if** t−1>0 **and** d.s(t−1)= **true and** l.b.s.s(t)= **false then**8:  Conduct l.b.s and update l.b.s.s(t)= **true**9: **end if**10: **if** d.s(t+1)= **true and** r.b.s.s(t)= **false then**11:  Conduct r.b.s and update r.b.s.s(t)= **true**12: **end if**13: **if** $r^{\left(t\right)}=D$ **and** d.s(t)= **false then**14:  Conduct decoding and  update d.s(t)= **true**, t=t−115:  **Go to** 316: **else**17:  **if** l.b.s.s(t)= **false and** t−1>0  **and** d.s(t−1)= **false then**18:   Conduct co.cs to obtain ch.cs with length ϕ19:   **if** d.s(ch.cs)= **true then**20:    Conduct decoding and    update d.s(t−ϕ+1:t)= **true**, t=t−ϕ21:    **Go to** 322:   **end if**23:  **end if**24: **end if**25:
**end if**
26:Wait to receive the codeword combinations of chunk i+1


### 5.3. Design with Applied Overlapped Chunked Code

The design of the applied OCC for multi-source multi-relay, i.e., the design of OCC/CF, is to determine an allocation $\left[\left(\mu_{1},\gamma_{1}\right),\left(\mu_{2},\gamma_{2}\right),\cdots ,\left(\mu_{K},\gamma_{K}\right)\right]$ with the desired $p_{d\mathrm{eff}}$ or with the desired effective channel efficiency $\eta_{\mathrm{eff}}$. For convenience, this paper takes *M* as the maximum chunk size, i.e., $M\geq \max\{d_{k},1\leq k\leq K\}$ and *M* can provide $\min\{\theta_{k},1\leq k\leq K\}>0$. If $N_{k}$ is the total number of blocks for source *k*, the number of chunks that contain the blocks of all sources is equal to $\min\left\{\left\lfloor \frac{N_{k}}{\mu_{k}}\right\rfloor ,1\leq k\leq K\right\}$.

The finite number of chunks for the applied OCC might cause high overhead in single-flow transmission if compared with the other codes such as in the works of [16,17,18,30], which have a rateless feature. However, the design of the applied OCC in a multi-source multi-relay network might be simpler if compared with the other designs that determine the probability distribution of chunk size for all sources, for example. By taking the design of BATs codes [17] as an example, $d_{k}$ is selected according to a determined degree distribution $\Psi_{k}=\left\{\psi_{0},\psi_{1},\cdots ,\psi_{M}\right\}$ for each chunk. Determining $\Psi_{k}$ for all *k* must consider the outputs of $p_{k}$, $p_{d}$ and $p_{d\mathrm{eff}}$, while $\Psi_{k}$ for all *k* needs to satisfy $\Psi_{1}*\Psi_{2}*\cdots *\Psi_{K}=\Psi$ [31], where Ψ is the degree distribution of $D=\sum_{k=1}^{K}d_{k}$ and sign ∗ refers to the discrete-time convolution operation. It becomes more complicated to determine $\Psi_{k}$ for all *k* when *K* is large.

For the design of OCC/CF, $d_{k}$ is fixed for all chunks except for the first chunk. Thus, there are $1+2+\cdots +M=\frac{M\left(M+1\right)}{2}$ candidates of $\left(\mu_{k},\gamma_{k}\right)$ for source *k* and ${\left[\frac{M\left(M+1\right)}{2}\right]}^{K}$ candidates of $\left[\left(\mu_{1},\gamma_{1}\right),\left(\mu_{2},\gamma_{2}\right),\cdots ,\left(\mu_{K},\gamma_{K}\right)\right]$ for all sources. It would be less if fixing $d_{k}=M$ and varying only $\gamma_{k}$ for all *k*, as in the previous work of this paper [1], where there are only *M* candidates for each source and $M^{K}$ candidates for all sources. However, the decoding complexity and the storage overhead at the destination might be high. For this work, the chunk size for each source is not large and bounded by *M*; however, it can be large, as in the work of [14]. A larger chunk size with a large number of overlapped blocks can improve the decodability of all chunks, but it can cause high computational complexity, especially decoding complexity and storage overhead at the destination. An undecodable chunk needs to wait for several new received chunks to start decoding, i.e., ϕ is large. For example, if taking μ=14, γ=18, $\bar{\rho }=15$ and M<D, where D=μ+γ=32, then there are no chunks that can be decoded by themselves, i.e., r=D without using b.s or co.cs. The decoding process can only start by co.cs, where ϕ at least satisfies:

(12) $$\phi \cdot \bar{\rho }\geq \phi \cdot \mu +\gamma ,$$

hence, ϕ≥18.

With a large chunk size, the decoding process rarely starts with back-substitution, i.e., the ch.cs with length ϕ=1. The decoding process only start with co.cs with large ϕ. In this work, the length ϕ is bounded for the purpose of low decoding complexity by providing more opportunities to conduct b.s and to reduce latency, as an undecodable chunk needs to wait to become decodable.

## 6. Estimation of Decodability

### 6.1. Overview

In order to select an appropriate allocation $\left[\left(\mu_{1},\gamma_{1}\right),\left(\mu_{2},\gamma_{2}\right),\cdots ,\left(\mu_{K},\gamma_{K}\right)\right]$ for the desired purpose, the estimation of $p_{d\mathrm{eff}}$ (also $\eta_{\mathrm{eff}}$) is done for each possible allocation. The estimations of $p_{k}$ and $p_{d}$ are conducted separately for each possible allocation. Then, $p_{d\mathrm{eff}}$ is determined by (11), and $\eta_{\mathrm{eff}}$ is determined by:

(13) $$\eta_{\mathrm{eff}}=\mu \cdot p_{d\mathrm{eff}}.$$

In the previous work of this paper [1], the estimation was done by conducting a simulation to obtain the performance in $p_{d}$ and $p_{k}$ of all allocations. Alternatively, in this paper, the estimation is done by conducting table lookup and the accumulative sum of the probabilities that ch.cs with the maximum length $\phi_{\max}$ are decodable for all possible combinations of $\left[r^{i-\phi +1},r^{i-\phi +2},\cdots ,r^{i}\right]$ and $\left[\theta_{k}^{i-\phi +1},\theta_{k}^{i-\phi +2},\cdots ,\theta_{k}^{i}\right]$ to determine $p_{d}$ and $p_{k}$, respectively, using $\rho \left(r\right)$ and $\lambda \left(\theta_{k}\right)$, respectively, for $\phi \in \{1,2,\cdots ,\phi_{\max}\}$ and i>1. For convenience, only the estimation of $p_{d}$ is described, and the estimation of $p_{k}$ for all *k* can be done similarly.

At the start, it is assumed that an OCC with the fashion as in Figure 6 is applied from a sender to a receiver in single-flow transmission, and $\rho \left(r\right)$ with $r\in \{1,2,\cdots ,D_{\max}\}$ is the obtained empirical rank distribution. The chunk size *D* is selected from the range value of *r*, then the maximum number of linearly independent codeword combinations (coded blocks) received per chunk becomes *D*. $\rho \left(r\right)$ is updated to $\rho^{\prime }\left(r^{\prime }\right)$ where $r^{\prime }\in \left\{1,2,\cdots ,D\right\}$. This paper estimates $\rho^{\prime }\left(r^{\prime }\right)$ from $\rho \left(r\right)$ by:

(14) $$\rho^{\prime }\left(r^{\prime }\right)=\left\{\begin{array}{cc} \rho \left(r\right), & \mathrm{if}\;r^{\prime }\in \left\{1,2,\cdots ,D-1\right\}.\\ \sum_{r=D}^{D_{\max}}\rho \left(r\right), & \mathrm{if}\;r^{\prime }=D. \end{array}\right.$$

For the estimation and for convenience, another four back-substitution states for a chunk are defined as below:

(i) not back-substituted state (n.b.s.s): equivalent to the event that l.b.s.s and r.b.s.s for a chunk are all false;
(ii) half back-substituted state (h.b.s.s): equivalent to the event that one of l.b.s.s and r.b.s.s is true;
(iii) full back-substituted state (f.b.s.s): equivalent to the event that l.b.s.s and r.b.s.s are all true;
(iv) quasi-half back-substituted state (q.b.s.s): equivalent to n.b.s.s in the b.s process and equivalent to h.b.s.s in the co.cs process.

n.b.s.s$\left(r^{\prime }\right)$, for example, refers to a chunk that has $r^{\prime }$ linearly independent coded blocks, and its state is n.b.s.s. $\varrho_{n}\left(r^{\prime }\right)$, $\varrho_{h}\left(r^{\prime }\right)$, $\varrho_{q}\left(r^{\prime }\right)$ and $\varrho_{f}\left(r^{\prime }\right)$ denote the probabilities that a chunk has $r^{\prime }$ linearly independent coded blocks and has n.b.s.s, h.b.s.s, f.b.s.s and q.b.s.s, respectively. They satisfy the condition below.

(15) $$\sum_{r^{\prime }=1}^{D}\left[\varrho_{n}\left(r^{\prime }\right)+\varrho_{h}\left(r^{\prime }\right)+\varrho_{q}\left(r^{\prime }\right)+\varrho_{f}\left(r^{\prime }\right)\right]=1.$$

Initially, $\varrho_{n}\left(r^{\prime }\right)=\rho^{\prime }\left(r^{\prime }\right)$ and $\varrho_{h}\left(r^{\prime }\right)=\varrho_{q}\left(r^{\prime }\right)=\varrho_{n}\left(r^{\prime }\right)=0$ for $r^{\prime }\in \left\{1,2,\cdots ,D\right\}$ are given. The estimation here is to update $\varrho_{n}\left(r^{\prime }\right)$, $\varrho_{h}\left(r^{\prime }\right)$, $\varrho_{q}\left(r^{\prime }\right)$ and $\varrho_{f}\left(r^{\prime }\right)$ according to the decoding process for all values of $r^{\prime }$. If $\rho_{d}$ denotes the probability distribution of the number of linearly independent coded blocks in a chunk after conducting the updating process, then:

(16) $$\rho_{d}\left(r^{\prime }\right)=\varrho_{n}\left(r^{\prime }\right)+\varrho_{h}\left(r^{\prime }\right)+\varrho_{q}\left(r^{\prime }\right)+\varrho_{f}\left(r^{\prime }\right),\mathrm{for}\;r^{\prime }\in \left\{1,2,\cdots ,D\right\}.$$

In the updating process, the chunk with n.b.s.s, h.b.s.s and q.b.s.s is active, i.e., $\varrho_{n}\left(r^{\prime }\right)$, $\varrho_{h}\left(r^{\prime }\right)$ and $\varrho_{q}\left(r^{\prime }\right)$ are used to conduct the updating process, and the chunk with f.b.s.s is inactive, i.e., $\varrho_{f}\left(r^{\prime }\right)$ cannot be used to conduct the updating process and is only used in determining $\rho_{d}$. The updating process is to try transforming n.b.s.s$\left(r^{\prime }\right)$ for all $r^{\prime }$ to the chunks with other states, i.e., to make $\varrho_{n}\left(r^{\prime }\right)$ tend to zero for all $r^{\prime }$. At the end of the updating process, $p_{d}$ is obtained by taking $p_{d}=\rho_{d}\left(D\right)$.

The updating process is divided into two parts: b.s and co.cs, which are for ϕ=1 and for $2\leq \phi \leq \phi_{\max}$, respectively. This paper assumes that the estimation of decodability is done at the destination. The destination informs about the desired allocation to the sources via feedback.

### 6.2. Combination of Chunks

A ch.cs with $C_{c}$ as in Figure 7 with length ϕ, where $2\leq \phi \leq \phi_{\max}$, is considered. The rank array of $C_{c}$ is $\left[r^{\prime (i-\phi +1)},r^{\prime (i-\phi +2)},\cdots ,r^{\prime (i)}\right]$, where $r^{\prime (t)}$ is the rank of $C^{\left(t\right)}$ for t∈{i−ϕ+1,i−ϕ+2,⋯,i}. A combination of $\left[r^{\prime (i-\phi +1)},r^{\prime (i-\phi +2)},\cdots ,r^{\prime (i)}\right]$ for a ch.cs (simply combination for convenience) with length ϕ is considered as a possible combination to be taken into account in the estimation if it satisfies:

(17) $$\sum_{t=i-\phi +1}^{i}r^{\prime (t)}\geq \phi \mu +\gamma ,$$

and it does not contain any possible combination with length $\phi^{\prime }$ inside, where $\phi^{\prime }\in \left\{1,2,\cdots ,\phi -1\right\}$. The ch.cs corresponding to a possible combination is decodable if $\mathrm{rank}\left(C_{c}\right)=\phi \cdot \mu +\gamma$. $p_{s}$ denotes the probability that a combination can make the correspondent ch.cs decodable and $q_{s}=1-p_{s}$. All possible combinations and their $p_{s}$ are obtained by conducting a computation in MATLAB in this work and known by the destination where table lookup is done while doing the estimation.

Based on three different locations of a chunk in a ch.cs, the other three probabilities are defined as below.

(i) $\varrho_{b}\left(r^{\prime }\right)$: the probability that a chunk that has $r^{\prime }$ linearly independent coded blocks can play the role of the beginning chunk, i.e., t=i. The beginning chunk can be with n.b.s.s or h.b.s.s or q.b.s.s.
(ii) $\varrho_{i}\left(r^{\prime }\right)$: the probability that a chunk that has $r^{\prime }$ linearly independent coded blocks can play the role of the intermediate chunk, i.e., t∈{i−ϕ+2,i−ϕ+3,⋯,i−1}. The intermediate chunk only can be with n.b.s.s. Hence, $\varrho_{i}\left(r^{\prime }\right)=\varrho_{n}\left(r^{\prime }\right)$.
(iii) $\varrho_{e}\left(r^{\prime }\right)$: the probability that a chunk that has $r^{\prime }$ linearly independent coded blocks can play the role of the ending chunk, i.e., t=i−ϕ+1. The ending chunk can be with n.b.s.s, or h.b.s.s, or q.b.s.s. The beginning chunk and the ending chunk have a similar property because the combinations are reversible. Thus, only the beginning chunk is studied, and $\varrho_{e}\left(r^{\prime }\right)=\varrho_{n}\left(r^{\prime }\right)+\varrho_{h}\left(r^{\prime }\right)+\varrho_{q}\left(r^{\prime }\right)$ is taken.

Chunk *t* is decodable, i.e., ch.cs that contains chunk *t* and t∈{i−ϕ+1,⋯,i} is decodable, by probability $p_{c}$, which is:

(18) $$p_{c}=p_{s}\varrho_{b}\left(r^{\prime (i)}\right)\varrho_{e}\left(r^{\prime (i-\phi +1)}\right)\prod_{t=i-\phi +2}^{i-1}\varrho_{i}\left(r^{\prime (t)}\right).$$

When the beginning chunk t=i is focused on, it must have $r^{\prime (t)}\geq \mu +1$, as in the case that one of $l.b.s.s\left(t\right)$ and $r.b.s.s\left(t\right)$ is true in Algorithm 2. For example, by taking D=18 and γ=4, a combination with rank array $\left[16,14,14,17\right]$ is a possible combination where $r^{\prime (t)}=16$ and $p_{s}\approx 0.9663$. However, the combination with rank array $\left[16,15,14,17\right]$ is not a possible combination because it contains a possible combination with rank array $\left[15,14,17\right]$.

When the intermediate chunk t∈{i−ϕ+2,⋯,i−1} is focused on, it must have $r^{\prime (t)}\geq \mu -\gamma +2$, as in the case that $l.b.s.s\left(t\right)$ and $r.b.s.s\left(t\right)$ are all false in Algorithm 2. However, for $r^{\prime (t)}>\mu$, the possible combinations that have the same rank arrays as those of the possible combination when focusing on the beginning chunk with the same value of $r^{\prime (t)}$ are not included. In this estimation, the possible combinations that are both available while focusing on the beginning chunk and the intermediate chunk and have the same elements of the rank array, and the same focused chunk with n.b.s.s is applied once in the updating process with co.cs. This is because there is no constraint on the order of chunks in a ch.cs for Relation (18). For example, by taking D=18 and γ=4, two combinations with rank array $\left[16,13,15,16\right]$ focusing on the chunk with $r^{\prime (t)}=13$ and with rank array $\left[16,15,16\right]$ focusing on the chunk with $r^{\prime (t)}=15$ are used because combinations with rank arrays $\left[13,16,15,16\right]$ focusing on the chunk with $r^{\prime (t)}=13$ and with rank array $\left[15,16,16\right]$ focusing on the chunk with $r^{\prime (t)}=15$ are not possible combinations. However, the combination with rank array $\left[16,13,15,16\right]$ focusing on the chunk with $r^{\prime (t)}=15$ is not a possible combination because the combination with rank array $\left[15,16,13,16\right]$ focusing on the chunk with $r^{\prime (t)}=15$ is a possible combination.

The purpose of introducing q.b.s.s is described by the following example. By taking D=18 and γ=3, a combination with rank array $\left[17,16,14,17\right]$ has $p_{s}\approx 0.9473$. However, it is not a possible combination since the combination with rank array $\left[17,16\right]$ is a possible combination with $p_{s}\approx 0.8369$. Then, no matter how the rest of the decoding process (with b.s) is done, the combination with rank array $\left[17,16,14,17\right]$ is decodable with $p_{s}$ less than 0.8369, which is lower than the real value. Hence, in order to fix this problem, q.b.s.s is introduced by assuming that the beginning chunk with n.b.s.s of an undecodable possible combination (with a probability of $q_{c}=q_{s}\varrho_{b}\left(r^{\prime (i)}\right)\varrho_{e}\left(r^{\prime (i-\phi +1)}\right)\prod_{t=i-\phi +2}^{i-1}\varrho_{i}\left(r^{\prime (t)}\right)$) becomes a chunk with $r^{\prime (i)}=D-1$, but no longer with n.b.s.s, and with a state like h.b.s.s during the updating process with co.cs and with a state like n.b.s.s during the updating process with b.s. After introducing q.b.s.s, the ch.cs with the rank array $\left[17,16,14,17\right]$ is decodable with a probability around 0.9498, which is close to the real value.

The relationship (transition) between four states of a chunk for the updating process with co.cs is shown in Table 2, where $p_{t}$ refers to the transfer probability.

The updating process with co.cs is done by applying the relationship in Table 2 and by following the concept of same increment same decrement, i.e., the decrement of the probability of a state (n.b.s.s or h.b.s.s or q.b.s.s) at a value of $r^{\prime }$ is the increment of probability of another state at the same value of $r^{\prime }$ or at the others. For example, by taking D=18, γ=3, q=7, $\varrho_{n}\left(r^{\prime }\right)=\left\{0.1,0.2,0.4,0.3\right\}$ and $\varrho_{h}\left(r^{\prime }\right)=\varrho_{q}\left(r^{\prime }\right)=\varrho_{f}\left(r^{\prime }\right)=\left\{0,0,0,0\right\}$ for $r^{\prime }\in \left\{15,16,17,18\right\}$ and by considering a chunk with n.b.s.s and $r^{\prime }=15$ in a possible combination with rank array $\left[16,15,17\right]$, then $p_{s}\approx 0.8166$ and $p_{c}=0.0065$ are obtained. Then, $\varrho_{n}\left(15\right)$ decreases by $p_{c}$, and $\varrho_{f}\left(18\right)$ increases by $p_{c}$. The updating process with co.cs can be done repeatedly for a number of iterations. $\varrho_{n}\left(r^{\prime }\right)$, $\varrho_{h}\left(r^{\prime }\right)$, $\varrho_{q}\left(r^{\prime }\right)$ and $\varrho_{f}\left(r^{\prime }\right)$ for all values of $r^{\prime }$ are updated after an iteration of the updating process with co.cs and are used for the next iteration.

### 6.3. Back-Substitution

The work in [28] provides the probability that $\gamma^{\prime }$ recovered (overlapped) blocks successfully help undecodable chunk *t* that has already had $r^{\prime (t)}$ linearly independent coded blocks turn into a decodable chunk, where $r^{\prime (t)}+\gamma^{\prime }\geq D$ and $\gamma^{\prime }\in \left\{\gamma ,2\cdot \gamma \right\}$. In this estimation, the increment of the number of linearly independent coded blocks in an undecodable chunk after conducting b.s by using $\gamma^{\prime }$ recovered blocks is also considered. In addition, the probability distribution of the increment is used.

In order to obtain these data, a computation in MATLAB is conducted to obtain the rank probability distribution of matrix $C^{\left(t\right)}\in F_{q}^{D\times r^{\prime (t)}}$ with $\gamma^{\prime }$ rows eliminated, i.e., a $\left(D-\gamma^{\prime }\right)\times r^{\prime (t)}$ matrix; hence, the obtained rank $r_{m}^{\left(t\right)}$ has range of values $\left\{0,1,\cdots ,r_{\max}^{\prime (t)}\right\}$, where $r_{\max}^{\prime (t)}=\min\left\{D-\gamma^{\prime },r^{\prime (t)}\right\}$. The probability distribution of $r_{m}^{\left(t\right)}$ is denoted by $\rho_{m}\left(r_{m}^{\left(t\right)}\right)$. Since the initial rank of $C^{\left(t\right)}$ is $r^{\prime (t)}$, then the probability distribution of the rank of the $\left(D-\gamma^{\prime }\right)\times r^{\prime (t)}$ matrix, denoted by $\rho_{m}^{\prime }\left(r_{m}^{\prime (t)}\right)$ where $r_{m}^{\prime (t)}\in \left\{r_{\min}^{\prime (t)},\cdots ,r_{\max}^{\prime (t)}\right\}$ and $r_{\min}^{\prime (t)}=\max\left\{0,r^{\prime (t)}-\gamma^{\prime }\right\}$, is approximately obtained by:

(19) $$\rho_{m}^{\prime }\left(r_{m}^{\prime (t)}\right)=\left\{\rho_{m}\left(r_{\min}^{\prime (t)}\right),\cdots ,\rho_{m}\left(r_{\max}^{\prime (t)}\right)\right\}\cdot \frac{\sum_{r_{m}^{\left(t\right)}=0}^{r_{\max}^{\prime (t)}}\rho_{m}\left(r_{m}^{\left(t\right)}\right)}{\sum_{r_{m}^{\left(t\right)}=r_{\min}^{\prime (t)}}^{r_{\max}^{\prime (t)}}\rho_{m}\left(r_{m}^{\left(t\right)}\right)}.$$

The rank increment from the aid of $\gamma^{\prime }$ overlapped blocks to undecodable chunk *t* is denoted by $r_{i}^{\left(t\right)}$; hence, $r_{i}^{\left(t\right)}=r_{m}^{\prime (t)}+\gamma^{\prime }-r^{\prime (t)}$, where $r_{i}^{\left(t\right)}\in \left\{0,1,\cdots ,\gamma^{\prime }\right\}$. If $\rho_{i}\left(r_{i}^{\left(t\right)}\right)$ is the probability distribution of $r_{i}^{\left(t\right)}$, then $\rho_{i}\left(r_{i}^{\left(t\right)}\right)=\rho_{m}^{\prime }\left(r_{m}^{\prime (t)}\right)$. $p_{bs}=\varrho_{n}\left(D\right)+\varrho_{h}\left(D\right)$ is taken as the fraction (probability) of the decoded chunks that can be used for b.s, and $q_{bs}=1-p_{bs}$. For the case that only half b.s (l.b.s or r.b.s) is executable, then $\gamma^{\prime }=\gamma$. If full b.s is executable, then $\gamma^{\prime }=2\cdot \gamma$. The transition table between four chunk states for the updating process with b.s is shown in Table 3. The focused chunk *t* has $C^{\left(t\right)}$ with rank $r^{\prime (t)}$.

The updating process with b.s is done by applying the relationship of four chunk states shown in Table 3 and by following the concept of the same increment same decrement as in the updating process with co.cs. The updating process with b.s can be done repeatedly as with co.cs. After the end of each iteration of the updating process with b.s, $p_{bs}$ is added to $\varrho_{f}\left(D\right)$, and it is updated according to the updated value of $\varrho_{h}\left(D\right)$ because $\varrho_{n}\left(D\right)$ becomes zero after the first iteration of the updating process with b.s.

### 6.4. Decoding Complexity

From the work in [17], to encode a block by applying RLNC from $d_{k}$ input blocks required $O\left(T\cdot d_{k}\right)$ finite field operation, where T is the number of symbols per block. By applying Gaussian elimination for the decoding process, decoding a chunk with size $d_{k}$ requires $O\left(d_{k}^{2}+T\cdot d_{k}\right)$ finite field operations per block on average. Since decoding complexity is more significant than encoding complexity, thus only decoding complexity is discussed in this paper.

With the applied decoding scheme, the main key to look at the decoding complexity is the mean length of ch.cs, $\bar{\phi }$. With OCC/CF, it is hard to estimate $\bar{\phi }$ since a successful b.s or co.cs does not depend on the selection of $\left[D,\gamma \right]$ alone, but actually on the selection of $\left[\left(\mu_{1},\gamma_{1}\right),\left(\mu_{2},\gamma_{2}\right),\cdots ,\left(\mu_{K},\gamma_{K}\right)\right]$. However, this paper employs the OCC designed using $\rho \left(r\right)$, i.e., the selection of $\left[D,\gamma \right]$, to estimate $\bar{\phi }$ since $p_{d}\geq p_{d\mathrm{eff}}$ and the obtained value of $\bar{\phi }$ might rely on the way of conducting the estimation.

$\varrho_{\phi }\left(\phi \right)$ denotes the probability (faction) that a ch.cs with length ϕ is successfully decoded. $\varrho_{\phi }\left(\phi \right)$ for $2\leq \phi \geq \phi_{\max}$ can be estimated or collected along with the process of updating probabilities with co.cs, i.e., the increase in $\varrho_{h}\left(D\right)$ and in $\varrho_{f}\left(D\right)$ for a considered ϕ is the increase in $\varrho_{\phi }\left(\phi \right)$. In addition, $\varrho_{\phi }\left(1\right)$ can be obtained by $p_{d}-\sum_{\phi =2}^{\phi_{\max}}\varrho_{\phi }\left(\phi \right)$. Hence, $\bar{\phi }$ can be obtained by:

(20) $$\bar{\phi }=\frac{\sum_{\phi =1}^{\phi_{\max}}\phi \cdot \varrho_{\phi }\left(\phi \right)}{p_{d}}.$$

Therefore, while employing OCC/CF, for each successfully decoding, it would need $O\left({\bar{\phi }}^{2}\cdot D^{2}+T\cdot \bar{\phi }\cdot D\right)$ finite field operations per block. It would be lower since $C_{c}$ is an approximately sparse matrix, as shown in Figure 7.

### 6.5. Obtaining the Estimated Performance

The process of updating probabilities with co.cs and b.s above can be done repeatedly and also alternately. Since there are μ innovative blocks per chunk, then chunk *t* with $r^{\prime (t)}>\mu$ can help the undecodable chunks to decode. This paper considers that applying the updating process with b.s first might make the value of $\varrho_{n}^{\prime }\left(r^{\prime (t)}\right)$ where $r^{\prime (t)}$ is close to *D* tend to zero early and then might make the other $\varrho_{n}^{\prime }\left(r^{\prime (t)}\right)$ hardly tend to zero via the updating process. Therefore, this paper applies the updating process with co.cs first.

By taking the overlapping fashion of the applied OCC as the pseudo-reference, the updating process with co.cs is applied for one iteration, then the updating process with b.s is applied for $\phi_{\max}$ iterations. If *N* is the total number of original blocks, then there are approximately $N_{\mathrm{ch}}=\left\lceil \frac{N}{\phi_{\max}}\right\rceil$ chains of chunks with length $\phi_{\max}$. It is assumed that one time of the updating process is the updating process with co.cs for one iteration and then with b.s for $\phi_{\max}$ iterations. Then, $N_{\mathrm{ch}}$ times of the updating process are needed such that the effect of the first ch.cs reaches the last ch.cs, and thus, $2\cdot N_{\mathrm{ch}}-1$ times of the updating process are needed such that this effect returns back to the first chunk. In this work, one round of the updating process is $2\cdot N_{\mathrm{ch}}-1$ times the updating process. In order to obtain the ultimate $\rho_{d}\left(r^{\prime }\right)$, the updating process is done for a number of rounds until the increment of obtained $p_{d}$ is lower than an assigned $\epsilon_{t}$, then the updating process terminates, and the ultimate $p_{d}$ is obtained. The channel efficient η is obtained by $\eta =\mu \cdot p_{d}$. The computational complexity for the estimation depends on $\epsilon_{t}$. It is higher when $\epsilon_{t}$ is smaller, but the accuracy might be higher.

**Figure 8:**
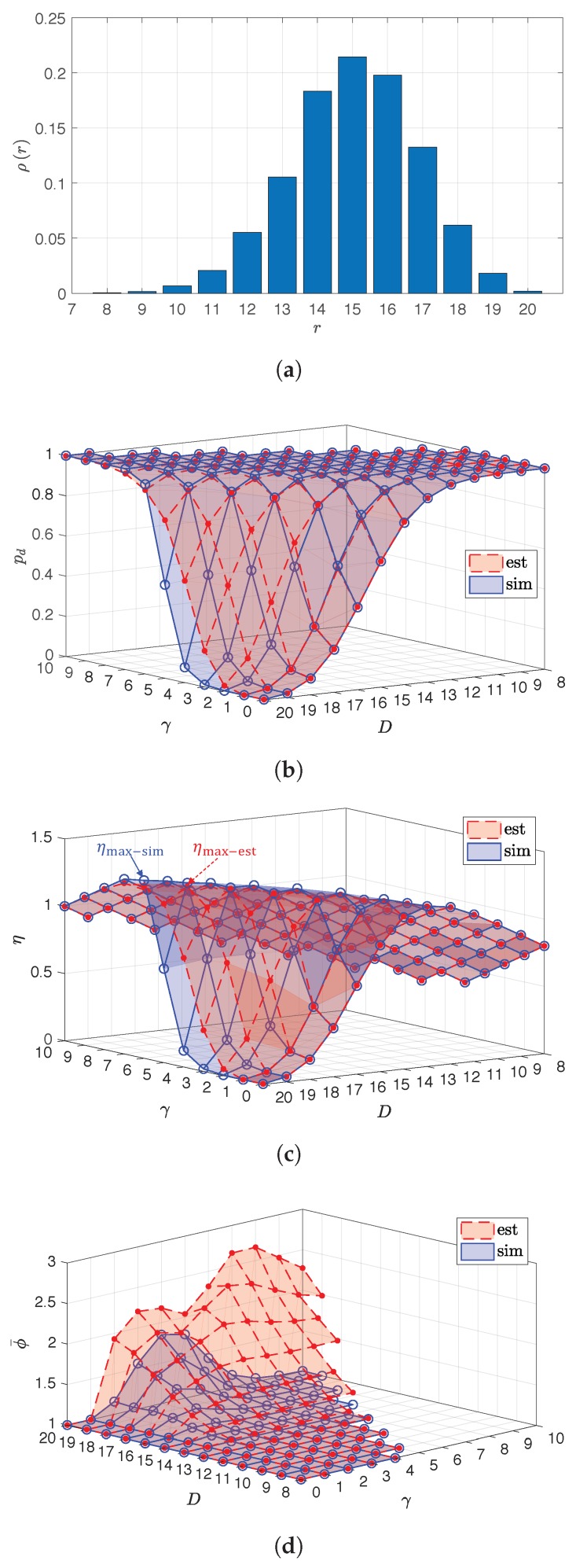
Comparison between the estimated $p_{d}$ and η (est) and the result from the simulation (sim) that were obtained from an empirical rank distribution $\rho \left(r\right)$. (**a**) Empirical rank distribution $\rho \left(r\right)$; (**b**) decodability; (**c**) channel efficiency; (**d**) mean length of ch.cs.

For example, by taking K=L=2, M=10, $\phi_{\max}=5$, ${\mathrm{SNR}}_{1,1}={\mathrm{SNR}}_{2,2}={\mathrm{SNR}}_{1,2}=35\mathrm{dB}$, ${\mathrm{SNR}}_{2,1}=15\mathrm{dB}$, $E_{8}/7E_{8}$ as NLC and by taking only the case that μ−γ>0, the empirical rank distribution $\rho \left(r\right)$, the estimated $p_{d}$ and the correspondent η are shown in Figure 8. The simulation result obtained with the same condition and by using the empirical rank distribution in Figure 8a is also shown in Figure 8b,c for comparison.

From Figure 8b, the estimation causes high deviation from the simulation result for the allocations $\left(\mu ,\gamma \right)$ that provide low $p_{d}$, e.g., lower than 0.9, and it causes low deviation for the allocations $\left(\mu ,\gamma \right)$ that provide high $p_{d}$. The error might be caused by the inaccuracy of the updated rank distribution $\rho^{\prime }$ or by the imperfectness of the process of updating probability with limited length of ch.cs. However, because the performance with high $p_{d}$ is preferred for this paper, thus the estimation applied in this paper is acceptable.

On the other hand, from Figure 8c, the maximum η from the estimation, $\eta_{\max-\mathrm{est}}$, is obtained by taking allocation $\left(14,5\right)$. However, the maximum η from the simulation, $\eta_{\max-\mathrm{sim}}$, is obtained by taking allocation $\left(14,6\right)$. Both allocations have the same μ, but different output $p_{d}$. From the work of [14,15], without limiting the length of ch.cs, larger γ with the same μ can provide higher $p_{d}$. Due to the estimation deviation, the allocation to provide $\eta_{\max}$ is not correctly given. However, during the application, the allocation providing $\eta_{\max-\mathrm{est}}$ can be switched to the other allocation with larger γ, but with the same μ to find out which allocation is more appropriate.

From Figure 8d, the estimated $\bar{\phi }$ is much larger than $\bar{\phi }$ from the simulation, since the process of updating probabilities with co.cs is executed first, which is different from the real fact that b.s should be conducted as soon as possible according to the decoding scheme described in Algorithm 4. Thus, $\varrho_{\phi }\left(\phi \right)$ is abnormally high for ϕ≥2, especially, when γ is large. However, the obtained results of $\bar{\phi }$ with the same μ=D−γ, but with different set values $\left(D,\gamma \right)$ from both estimation and simulation show that larger γ results in higher $\bar{\phi }$, hence higher decoding complexity. In addition, $\bar{\phi }$ from the estimation somehow can serve as $\bar{\phi }$ obtained in the worst case.

## 7. Performance Analysis

### 7.1. Examples of Allocations

This paper assumes that the fairness between sources is achieved if $\eta_{1}=\eta_{2}=\cdots =\eta_{K}$ where $\eta_{k}$ is the channel efficiency for source *k*. $\eta_{k}$ is defined as the ratio of the number of decoded blocks from source *k* to the number of time slots taken from the sources to the relays. If individually decoding is not considered, $\eta_{k}$ for all *k* only depends on $p_{d\mathrm{eff}}$. In this case, the fairness is achieved by taking $\mu_{1}=\mu_{2}=\cdots =\mu_{K}$.

By taking the data in Figure 8, Table 4 lists some allocations and their performances in $p_{d\mathrm{eff}}$ and $\eta_{\mathrm{eff}}$ from estimation and simulation. In addition, $\bar{\phi }$ is the average value of ϕ, and it is counted when a ch.cs with length ϕ≥1 is decoded. $\bar{\phi }$ represents the computational complexity of the decoding process.

From Table 4, Allocation 1 can provide the fairness between sources, but it cannot provide the highest channel efficiency, while Allocations 2 and 3 can provide the highest channel efficiency by estimation and simulation, respectively. The interchanged Allocation 3, i.e., Allocation 4, shows the affect of an unsuitable selection of $\left(\mu_{2},\gamma_{2}\right)$, which has low $p_{d\mathrm{eff}}$. Allocations 1–4 do not provide high $p_{d\mathrm{eff}}$. On the other hand, Allocation 5 can provide high $p_{d\mathrm{eff}}$, but not the highest channel efficiency. However, the decoding complexity is lower since $\bar{\phi }$ is smaller. If the difference between the provided channel efficiency and the highest channel efficiency is small, then this allocation can be applied instead if lower decoding complexity is required. Allocations 5–9 have the same μ, but different γ, which varies from 7–3. They show the outcome of different values of γ to $p_{d\mathrm{eff}}$ and $\bar{\phi }$. From the result in Table 4, larger γ provides higher $p_{d\mathrm{eff}}$, but higher decoding complexity.

On the other hand, the highest channel efficiency can be obtained by the precoding process at each source before employing OCC. However, this might cause additional decoding complexity and latency caused from re-ordering blocks after the decoding process. This paper assumes that there is no precoding overhead, i.e., the number of required received coded blocks is equal to the number of original blocks when the maximum channel efficiency is considered.

### 7.2. Impact of the Participation Factor of Each Source

From now on, this paper uses the term OCC as the applied OCC, which uses the allocation providing the highest channel efficiency. In addition, OCC${}^{\prime }$ refers to the applied OCC that uses the allocation providing the highest channel efficiency with condition $p_{d}\geq p_{\mathrm{thr}}$ or $p_{k}\geq p_{\mathrm{thr}}$. OCC/CF and OCC${}^{\prime }$/CF refer to the transmission schemes employing OCC and OCC${}^{\prime }$, respectively, before NLC in a multi-source multi-relay network. The decodability condition for OCC${}^{\prime }$/CF is $p_{d\mathrm{eff}}\geq p_{\mathrm{thr}}$.

**Figure 9:**
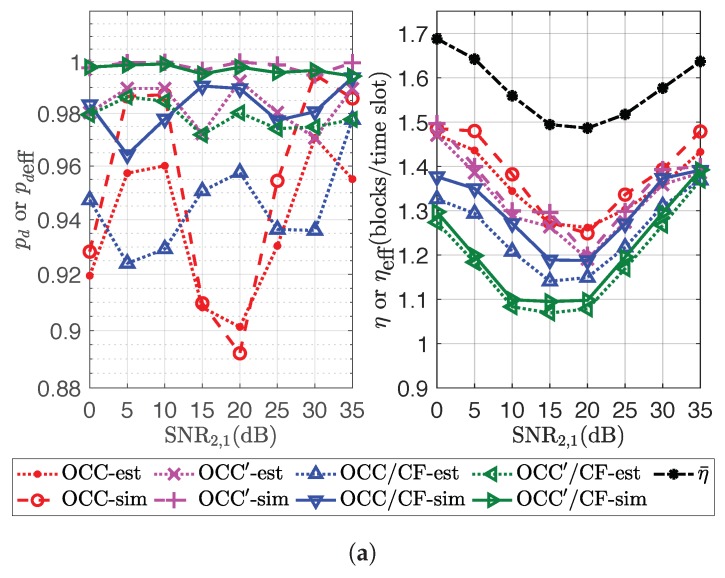

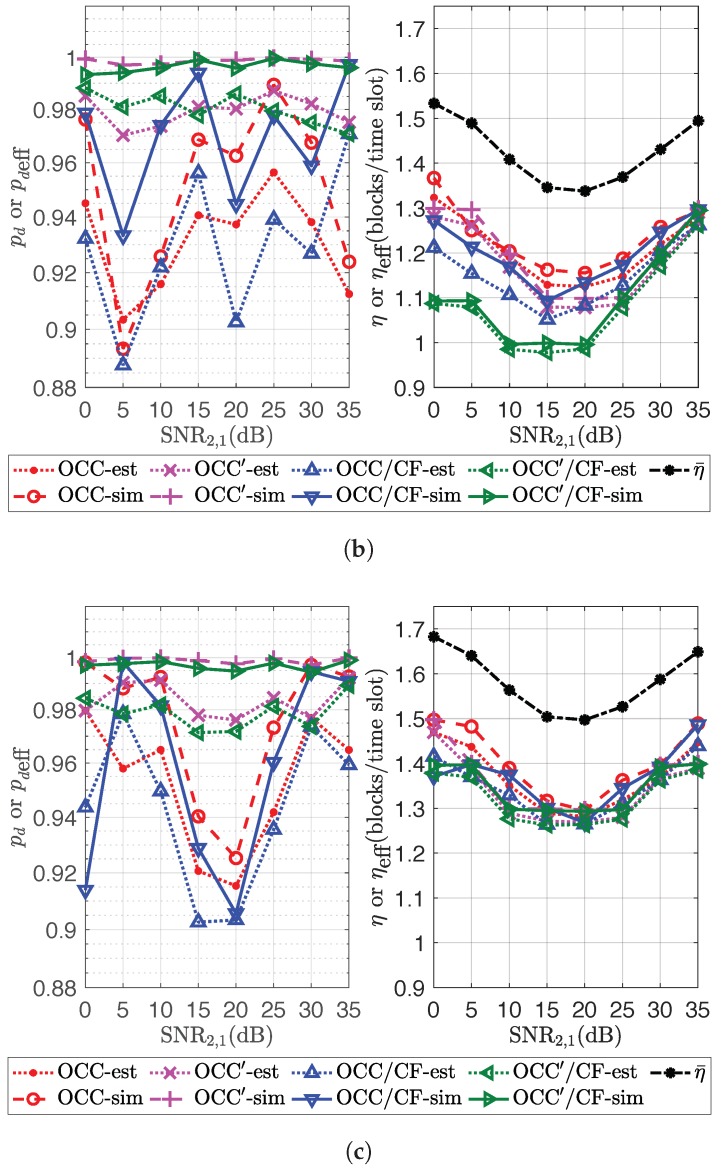
Decodability and channel efficiency of OCC/compute-and-forward (CF) and OCC${}^{\prime }$/CF from estimation and simulation. (**a**) ${\mathrm{SNR}}_{1,2}=5\mathrm{dB}$; (**b**) ${\mathrm{SNR}}_{1,2}=20\mathrm{dB}$; (**c**) ${\mathrm{SNR}}_{1,2}=35\mathrm{dB}$.

$\eta_{\max}$ denotes the maximum channel efficiency that can be provided by the applied OCC designed using $\rho \left(r\right)$. If individually decoding is not considered, from (11), the upper bound of $\eta_{\mathrm{eff}}$ is $\eta_{\max}$. As mentioned above, $\bar{\eta }=\frac{\bar{\rho }}{M}$ is the channel capacity or the upper bound of the channel efficiency for the transmission scheme employing OCC/CF from the sources to the destination. By taking K=L=2, M=10, $\phi_{\max}=5$, $E_{8}/7E_{8}$ as NLC, $p_{\mathrm{thr}}=0.97$, ${\mathrm{SNR}}_{1,1}={\mathrm{SNR}}_{2,2}=35\mathrm{dB}$, ${\mathrm{SNR}}_{1,2}\in \left\{5,20,35\right\}\mathrm{dB}$, ${\mathrm{SNR}}_{2,1}\in \left\{0,5,10,15,20,25,30,35\right\}\mathrm{dB}$. The performance of OCC/CF and OCC${}^{\prime }$/CF in decodability and channel efficiency from estimation (with postfix “-est”) and simulation (with postfix “-sim”) is shown in Figure 9.

From Figure 9, the estimated channel efficiencies of OCC/CF and OCC${}^{\prime }$/CF are around 97.95% and 98.34%, respectively, of those of the channel efficiency obtained from simulation on average. In addition, the gap between the channel efficiency of OCC and $\bar{\eta }$ represents the design overhead of applied OCC. From the simulation result, $\eta_{\max}$ is around 87.71% of $\bar{\eta }$ on average. The design overhead of OCC/CF should be the aggregate of the design overhead using $\rho \left(r\right)$ and $\lambda_{k}\left(\theta_{k}\right)$ for all *k*. The channel efficiency of OCC/CF and OCC${}^{\prime }$/CF is close to $\eta_{\max}$ when ${\mathrm{SNR}}_{1,2}$ or ${\mathrm{SNR}}_{2,1}$ is close to (as high as) ${\mathrm{SNR}}_{1,1}$ or ${\mathrm{SNR}}_{2,2}$. This is because, in this case, the participation factor of each source is dense around $\theta_{k}=d_{k}$. When $\lambda_{k}\left(d_{k}\right)$ is very dense, most of the allocations $\left(\mu_{k},\gamma_{k}\right)$ can provide $p_{k}$ close to one. Hence, the design of OCC/CF or OCC${}^{\prime }$/CF can only depend on $\rho \left(r\right)$.

In the case that ${\mathrm{SNR}}_{1,2}$ and ${\mathrm{SNR}}_{2,1}$ are low, the received combined coded blocks are almost plain coded blocks, i.e., $\beta_{lm}^{\left(i\right)}$ are almost in the form of unit vectors for all *l* and *m*. The design overheads of OCC/CF and OCC${}^{\prime }$/CF in this case should be the aggregate of the design overhead of OCCs using $\lambda_{k}\left(\theta_{k}\right)$ for all *k*. Since the participation factor of each source is not dense around $\theta_{k}=d_{k}$, the design overheads of OCC and OCC${}^{\prime }$ might be high, and higher than those of the prior case.

In order to make $\lambda_{k}\left(d_{k}\right)$ denser, in addition to forcing to obtain $\beta_{lm}^{\left(i\right)}$ without zero elements at each relay, improving the diversity of the received codeword combinations by increasing the number of participating relays or equipping more antennas at relays as in work of [8,9] might be a solution.

### 7.3. Reference Schemes

Because the original work in [7] did not consider the retransmission, a feedback-based transmission scheme is used as the reference scheme instead to evaluate the performance of OCC/CF and OCC${}^{\prime }$/CF, and it is called CF with protocol overhead (CF/PO) in this paper. For each round of CF/PO, each source applies NLC without OCC and needs feedback from the destination after sending a block to know which blocks have been decoded and which blocks need to be retransmitted. Feedback is forwarded by relays via an orthogonal channel. There is no decoding delay constraint, i.e., a source can transmit a new original block although the previous blocks of the other sources have not been decoded [6].

The protocol overhead (the transmission time of feedback and the loss of feedback) are taken into account. The feedback reception success rate is denoted by $p_{f}$, and the ratio of the transmission time of feedback to a slot time is denoted by $\tau_{f}$. $p_{f}$ is obtained by conducting a simulation where NLC is applied on a link from a source to a relay with the highest SNR via an orthogonal channel. Because it is hard to track the performance of CF/PO with varying $p_{f}$, only the performance with different values of $\tau_{f}$ is considered.

In addition to the channel efficiency, the transmission efficiency $\varepsilon_{t}$ is also considered to evaluate the performances of OCC/CF and OCC${}^{\prime }$/CF with a scheme called RLNC with orthogonal channel (RLNC/OC) where RLNC is applied before NLC at each source for the transmissions from the sources to the relays via an orthogonal channel. For each source, original blocks are grouped into disjoint chunks with *M* blocks per chunk. RLNC is applied within each chunk, and a feedback (ACK) is needed when a transmitted chunk is decodable. The protocol overhead is also considered, and it is assumed that feedback cannot be received instantaneously by the source to stop transmitting [32]. In this paper, $\varepsilon_{t}$ is defined as the ratio of the total number of decoded blocks to the total number of transmissions taken between the sources and the relays, while the transmission of feedback is also taken into account. In this paper, $\tau_{f}$ is assumed as the ratio of the length of feedback data to the length of payload data per block. The performance in transmission efficiency reflects the energy consumption of each scheme.

On the other hand, a transmission scheme employing LT code [20,30] at each source before NLC, called fountain code with CF (FC/CF), is also used as a reference scheme. The considered parameters of LT code are $c_{\mathrm{fc}}$ and $\delta_{\mathrm{fc}}$. In the case of single flow transmission with $N_{k}$ original blocks, the receiver can recover all blocks with probability $1-\delta_{\mathrm{fc}}$ if receiving $N_{k}+2\cdot \log_{e}\left(S_{\mathrm{fc}}/\delta_{\mathrm{fc}}\right)\cdot S_{\mathrm{fc}}$ coded blocks, where $S_{\mathrm{fc}}\equiv c_{\mathrm{fc}}\cdot \log_{e}\left(N_{k}/\delta_{\mathrm{fc}}\right)\cdot \sqrt{N_{k}}$.

In the CF/PO and FC/CF schemes, the decoding process is done for each source block transmission. The destination tries to decode if there are *K* linearly independent codeword combinations. If undecodable, the destination stores the undecodable blocks (after decoding using (2)) and waits for the next received codeword combinations.

Since feedback is not needed in OCC/CF, OCC${}^{\prime }$/CF and FC/FC, their transmission efficiency is 1/K times their channel efficiency. If $N_{\mathrm{dec}}$, $N_{\mathrm{fb}}$ and $N_{\mathrm{ts}}$ denote the total number of decoded blocks, the total number of feedback and the total number of time slots taken excluding the transmission time of feedback, respectively, then the channel efficiencies and the transmission efficiencies of CF/PO and RLNC/OC can be written as below.

(21) $$\eta_{\mathrm{eff}\_\mathrm{CF}/\mathrm{PO}}=\frac{N_{\mathrm{dec}}}{N_{\mathrm{ts}}+\tau_{f}\cdot N_{\mathrm{fb}}}.$$

(22) $$\varepsilon_{t\_\mathrm{CF}/\mathrm{PO}}=\frac{N_{\mathrm{dec}}}{K\cdot N_{\mathrm{ts}}+\tau_{f}\cdot N_{\mathrm{fb}}}.$$

(23) $$\eta_{\mathrm{eff}\_\mathrm{RLNC}/\mathrm{OC}}=\frac{N_{\mathrm{dec}}}{N_{\mathrm{ts}}}.$$

(24) $$\varepsilon_{t\_\mathrm{RLNC}/\mathrm{OC}}=\frac{N_{\mathrm{dec}}}{N_{\mathrm{ts}}+\tau_{f}\cdot N_{\mathrm{fb}}}.$$

## 8. Numerical Results and Discussion

This papers considers two scenarios to observe the performance of the transmission schemes employing OCC/CF and OCC${}^{\prime }$/CF by comparing with the reference schemes. The first scenario investigates the performance in a two-source two-relay network with an asymmetric channel state at relays, i.e., average SNRs of all links from all sources to a relay might be different, as used in Section 7.2. The second scenario considers a varying number of relays with a symmetric channel state, i.e., average SNRs of all links from all sources to a relay are the same, and the number of sources is fixed to two. The numerical results are obtained by conducting simulations taking $E_{8}/7E_{8}$ as NLC, M=10, $N_{k}=1000$ for all *k*, $c_{\mathrm{fc}}=0.01$, $\delta_{\mathrm{fc}}=0.01$, $\tau_{f}=0.05$ for RLNC/OC and $\tau_{f}\in \left\{0,0.05\right\}$ for CF/OF. The simulation frequency is 100 times. Each simulation terminates when there is at least source *k* having the rest of innovative blocks less than $\mu_{k}$ for OCC/CF, OCC${}^{\prime }$/CF and RLNC/OC and when all original blocks of at least one source are recovered for CF/PO and FC/CF.

**Figure 10:**
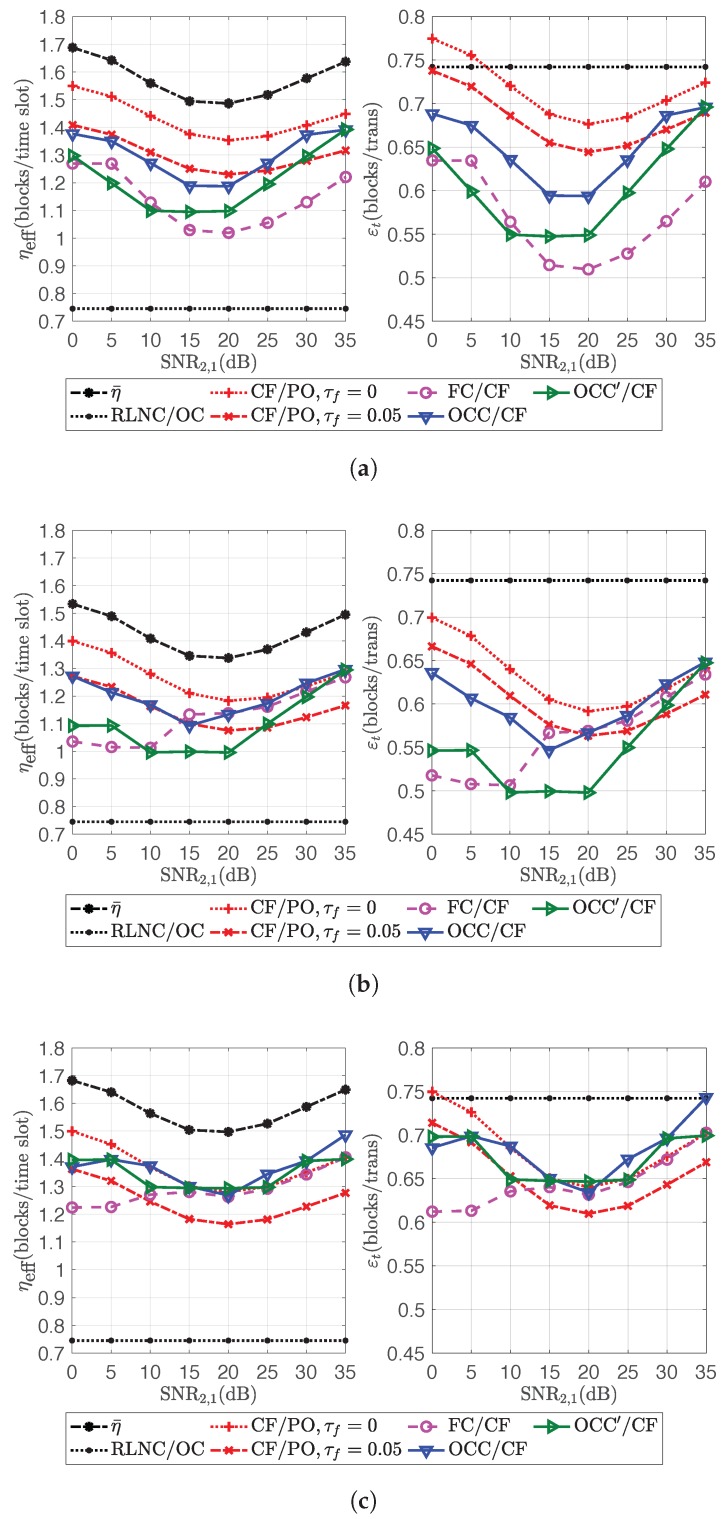
Channel efficiency and transmission efficiency for Scenario 1. (**a**) ${\mathrm{SNR}}_{1,2}=5\mathrm{dB}$; (**b**) ${\mathrm{SNR}}_{1,2}=20\mathrm{dB}$; (**c**) ${\mathrm{SNR}}_{1,2}=35\mathrm{dB}$.

**Figure 11:**
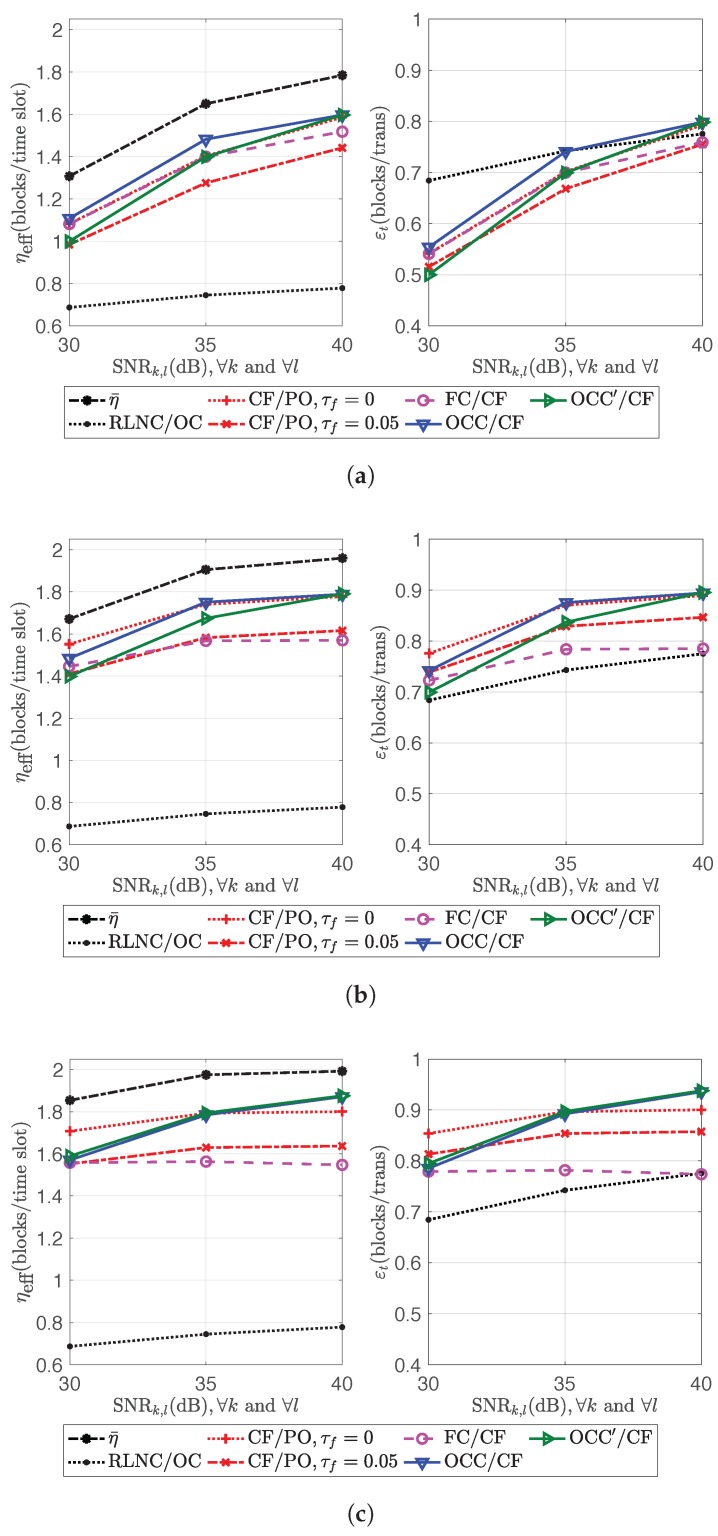
Channel efficiency and transmission efficiency for Scenario 2. (**a**) Two-source two-relay network; (**b**) two-source three-relay network; (**c**) two-source four-relay network.

The performances of OCC/CF and OCC${}^{\prime }$/CF in the first scenario are the same as in Figure 9. The second scenario takes ${\mathrm{SNR}}_{1,1}={\mathrm{SNR}}_{2,2}={\mathrm{SNR}}_{1,2}={\mathrm{SNR}}_{2,1}\in \left\{30,35,40\right\}\mathrm{dB}$ with correspondent $p_{f}\in \left\{0.8266,0.9021,0.9449\right\}$. The performances in channel efficiency and transmission efficiency of all schemes in Scenario 1 and 2 are shown in Figure 10 and Figure 11, respectively.

From Figure 10, OCC/CF and OCC${}^{\prime }$/CF increased channel efficiency by 72.98% and 63.28%, respectively, on average if comparing with RLNC/OC. For $\tau_{f}=0.05$, OCC/CF had a 4.71% increment of channel efficiency on average if comparing with CF/PO. However, OCC/CF and OCC${}^{\prime }$/CF provided higher channel efficiency than CF/PO only when ${\mathrm{SNR}}_{1,2}$ or ${\mathrm{SNR}}_{2,1}$ was similarly high as ${\mathrm{SNR}}_{1,1}$ or ${\mathrm{SNR}}_{2,2}$, i.e., when the participation factor of source *k* was dense around $\theta_{k}=d_{k}$ for all *k*. OCC/CF and OCC${}^{\prime }$/CF provided the increment of channel efficiency up to 16.41% and 13.41% if comparing with CF/PO for $\tau_{f}=0.05$. On the other hand, by comparing with FC/CF, OCC/CF had higher channel efficiency than FC/CF in almost all cases. The increment was around 9.36% on average. For OCC${}^{\prime }$/CF, because of the higher design overhead, it sometimes could not provide higher channel efficiency than FC/CF. There was only a 2.91% increment of channel efficiency on average if comparing with FC/CF. The issue of employing fountain code in this scenario was that the number of coded blocks to ensure the desired decodability was larger than the expected number, because some coded blocks from a source could not be extracted from the codeword combinations forwarded from the relays during each source block transmission.

On the other hand, the transmission efficiency of RLNC/OC was higher than the other schemes in all cases except CF/PO for $\tau_{f}=0$. Thus, OCC/CF and OCC${}^{\prime }$/CF had less of a chance to perform better than CF/PO when $\tau_{f}$ was small. The transmission efficiency of OCC/CF and OCC${}^{\prime }$/CF was 86.85% and 81.98% on average, respectively, of the transmission efficiency of RLNC/OC. It seems the performance of the proposed schemes was a trade-off between the increment of channel efficiency and the decrement of transmission efficiency if comparing with an orthogonal channel transmission scheme, e.g., RLNC/OC for this scenario. In order to improve the transmission efficiency of the proposed schemes, increasing the diversity of codeword combinations at relays, i.e., increasing the number of participating relays, was considered and discussed in Scenario 2.

From Figure 11, the channel efficiencies of OCC/CF and OCC${}^{\prime }$/CF increased when ${\mathrm{SNR}}_{k,l}$ increased, since the channel capacity $\bar{\rho }$ also increased with ${\mathrm{SNR}}_{k,l}$. OCC/CF and OCC${}^{\prime }$/CF performed similarly at high ${\mathrm{SNR}}_{k,l}$, and they had a chance to perform better than CF/PO for $\tau_{f}=0$ in the case of four relays at ${\mathrm{SNR}}_{k,l}=40\mathrm{dB}$ since the loss of feedback also impacted the performance of CF/PO. If comparing with CF/PO, OCC/CF and OCC${}^{\prime }$/CF increased channel efficiency up to 3.93% and 4.17%, respectively, for $\tau_{f}=0$, and up to 16.18% and 14.59%, respectively, for $\tau_{f}=0.05$. If comparing with RLNC/OC, OCC/CF and OCC${}^{\prime }$/CF increased channel efficiency up to 140.32% and 140.88%, respectively. OCC${}^{\prime }$/CF sometimes performed slightly better than OCC/CF, because of the estimation deviation. In addition, the channel efficiency of OCC/CF and OCC${}^{\prime }$/CF increased faster than that of FC/CF when ${\mathrm{SNR}}_{k,l}$ increased or the number of relays increased. This is because the overhead of fountain code was the same if the parameters $c_{\mathrm{fc}}$ and $\delta_{\mathrm{fc}}$ were fixed. In addition, it might have been because of the condition of stopping the simulation, i.e., all original blocks of a source were recovered, and those of the other source had not been all recovered, the channel efficiency of FC/CF did not increase when ${\mathrm{SNR}}_{k,l}$ increases or the number of relays increased, as shown in Figure 11c.

For the performance in transmission efficiency in Figure 11, OCC/CF and OCC${}^{\prime }$/CF had higher transmission efficiency than RLNC/OC when the number of relays was higher than the number of sources. However, employing more relays might have increased the complexity of the network such as how to select which relays to join, how to achieve time synchronization at all relays, etc.

On the other hand, the performance of OCC/CF and OCC${}^{\prime }$/CF could be improved, especially at low ${\mathrm{SNR}}_{1,2}$ and low ${\mathrm{SNR}}_{2,1}$ in the first scenario by applying decoding individually, but this might have increased the complexity of the decoding process if *K* were large.

## 9. Conclusions

This paper proposed a design of OCC that is applied before NLC in multi-source multi-relay networks, called OCC/CF. A decodability condition was provided for the design. This paper took an OCC with a contiguously overlapping fashion, but not a rounded-end fashion, to design OCC/CF. The decoding scheme and the estimation of designed OCC/CF are provided. The estimation is done for each allocation, i.e., the number of innovative blocks per chunk and the number of blocks taken from the previous chunk, to search for which allocation can provide the desired performance such as the highest channel efficiency, or the preferred decodability, or the acceptable decoding complexity. The estimation deviation is low when the decodability is sufficiently high. Since there are a limited number of chunks for the designed OCC/CF, the design overhead is high if comparing with channel capacity. From the numerical results, the advantage of OCC/CF over a feedback-based transmission scheme depends on the level of protocol overhead, i.e., the transmission time and the size of feedback, the feedback loss rate. The performance of OCC/CF, especially transmission efficiency when comparing with an orthogonal channel transmission, can be improved by increasing the number of relays. Future work is to consider decoding individually and the cooperation between feedback and OCC/CF for higher performance.

## Figures and Tables

**Table 1:** Specification of the simulation platform.

Term	Description
Processor	2.5-GHz Intel Core i7
Memory	16-GB 1600-MHz DDR3
Operating System	Mac OS
Software Tool	MATLAB

**Table 2:** Transition table between four chunk states for the updating process with the combination of chunks (co.cs). h.b.s.s, half back-substituted state; f.b.s.s, full back-substituted state; n.b.s.s, not back-substituted state; q.b.s.s, quasi-half back-substituted state.

Condition for Focused Chunk *t* with $r^{\prime (t)}$	From	To	$\varrho_{b}\left(r^{\prime (i)}\right)$	$p_{t}$
t=i; $r^{\prime (t)}\geq \mu +1$	h.b.s.s$\left(r^{\prime (t)}\right)$	f.b.s.s(D)	$\varrho_{h}\left(r^{\prime (t)}\right)$	$p_{c}$
t=i; $r^{\prime (i)}\geq \mu +1$	h.b.s.s$\left(r^{\prime (t)}\right)$	h.b.s.s$\left(r^{\prime (t)}\right)$	$\varrho_{h}\left(r^{\prime (t)}\right)$	$q_{c}$
i−ϕ+2≤t≤i−1; $r^{\prime (t)}\geq \mu -\gamma +2$	n.b.s.s$\left(r^{\prime (t)}\right)$	f.b.s.s(D)	$\varrho_{e}\left(r^{\prime (i)}\right)$	$p_{c}$
i−ϕ+2≤t≤i−1; $r^{\prime (t)}\geq \mu -\gamma +2$	n.b.s.s$\left(r^{\prime (t)}\right)$	n.b.s.s$\left(r^{\prime (t)}\right)$	$\varrho_{e}\left(r^{\prime (i)}\right)$	$q_{c}$
t=i; $r^{\prime (t)}\geq \mu +1$	n.b.s.s$\left(r^{\prime (t)}\right)$	h.b.s.s(D)	$\varrho_{n}\left(r^{\prime (t)}\right)$	$p_{c}$
t=i; $r^{\prime (t)}\geq \mu +1$	n.b.s.s$\left(r^{\prime (t)}\right)$	q.b.s.s(D−1)	$\varrho_{n}\left(r^{\prime (t)}\right)$	$q_{c}$
t=i; $r^{\prime (t)}=D-1$	q.b.s.s$\left(r^{\prime (t)}\right)$	h.b.s.s(D)	$\varrho_{q}\left(r^{\prime (t)}\right)$	$p_{c}$
t=i; $r^{\prime (t)}\geq \mu +1$	q.b.s.s$\left(r^{\prime (t)}\right)$	q.b.s.s$\left(r^{\prime (t)}\right)$	$\varrho_{q}\left(r^{\prime (t)}\right)$	$q_{c}$

**Table 3:** Transition table between four chunk states for the updating process with b.s.

Half or Full b.s or N/A	From	To	$p_{t}$
Half	n.b.s.s$\left(r^{\prime (t)}\right)$	h.b.s.s$\left(r^{\prime (t)}+r_{i}^{\left(t\right)}\right)$	$2\cdot p_{bs}\cdot q_{bs}\cdot \rho_{i}\left(r_{i}^{\left(t\right)}\right)$
Full	n.b.s.s$\left(r^{\prime (t)}\right)$	f.b.s.s$\left(r^{\prime (t)}+r_{i}^{\left(t\right)}\right)$	$p_{bs}^{2}\cdot \rho_{i}\left(r_{i}^{\left(t\right)}\right)$
N/A	n.b.s.s$\left(r^{\prime (t)}\right)$	n.b.s.s$\left(r^{\prime (t)}\right)$	$q_{bs}^{2}$
Half	h.b.s.s$\left(r^{\prime (t)}\right)$	f.b.s.s$\left(r^{\prime (t)}+r_{i}^{\left(t\right)}\right)$	$p_{bs}\cdot \rho_{i}\left(r_{i}^{\left(t\right)}\right)$
N/A	h.b.s.s$\left(r^{\prime (t)}\right)$	h.b.s.s$\left(r^{\prime (t)}\right)$	$q_{bs}$
Half	q.b.s.s$\left(r^{\prime (t)}\right)$	h.b.s.s$\left(r^{\prime (t)}+r_{i}^{\left(t\right)}\right)$	$2\cdot p_{bs}\cdot q_{bs}\cdot \rho_{i}\left(r_{i}^{\left(t\right)}\right)$
Full	q.b.s.s$\left(r^{\prime (t)}\right)$	f.b.s.s$\left(r^{\prime (t)}+r_{i}^{\left(t\right)}\right)$	$p_{bs}^{2}\cdot \rho_{i}\left(r_{i}^{\left(t\right)}\right)$
N/A	q.b.s.s$\left(r^{\prime (t)}\right)$	q.b.s.s$\left(r^{\prime (t)}\right)$	$q_{bs}^{2}$

**Table 4:** Some examples of allocations.

	Allocations				Estimation						Simulation		
**No.**	$\mu_{1}$	$\mu_{2}$	$\gamma_{1}$	$\gamma_{2}$	$p_{1}$	$p_{2}$	$p_{d}$	$p_{d\mathrm{eff}}$	$\eta_{\mathrm{eff}}$	$\bar{\phi }$	$p_{d\mathrm{eff}}$	$\eta_{\mathrm{eff}}$	$\bar{\phi }$
1	7	7	2	3	0.9982	0.9722	0.9206	0.8935	1.2509	2.1671	0.9107	1.2750	1.4702
2	8	6	2	3	0.9868	0.9935	0.9206	0.9026	1.2636	2.1671	0.9295	1.3013	1.4264
3	8	6	2	4	0.9868	0.9951	0.9152	0.8986	1.2580	2.3136	0.9341	1.3077	1.4820
4	6	8	3	2	0.9988	0.8309	0.9206	0.7641	1.0687	2.1671	0.6674	0.9344	1.6135
5	7	6	3	4	0.9975	0.9951	0.9756	0.9684	1.2589	2.6592	0.9991	1.2988	1.1812
6	7	6	2	4	0.9982	0.9951	0.9779	0.9714	1.2628	2.3758	0.9986	1.2982	1.1767
7	7	6	1	4	0.9989	0.9951	0.9710	0.9710	1.2623	2.0224	0.9938	1.2919	1.1679
8	7	6	0	4	0.9981	0.9951	0.9674	0.9710	1.2577	1.6220	0.9807	1.2749	1.1549
9	7	6	1	2	0.9989	0.9947	0.9697	0.9635	1.2525	1.2880	0.9833	1.2783	1.0815
